# Influenza A virus infection in turkeys induces respiratory and enteric bacterial dysbiosis correlating with cytokine gene expression

**DOI:** 10.7717/peerj.11806

**Published:** 2021-07-22

**Authors:** John M. Ngunjiri, Kara J.M. Taylor, Hana Ji, Michael C. Abundo, Amir Ghorbani, Mahesh KC, Chang-Won Lee

**Affiliations:** 1 Center for Food Animal Health, Ohio Agricultural Research and Development Center, Ohio State University, Wooster, OH, United States of America; 2 Department of Biology, University of Florida, Gainesville, FL, United States of America; 3 Department of Veterinary Preventive Medicine, College of Veterinary Medicine, Ohio State University, Columbus, OH, United States of America; 4 Center for Vaccines and Immunity, Abigail Wexner Research Institute at Nationwide Children’s Hospital, Columbus, OH, United States of America

**Keywords:** Respiratory microbiome, Dysbiosis, Proteobacteria, Avian influenza virus, Virus-induced dysbiosis

## Abstract

Turkey respiratory and gut microbiota play important roles in promoting health and production performance. Loss of microbiota homeostasis due to pathogen infection can worsen the disease or predispose the bird to infection by other pathogens. While turkeys are highly susceptible to influenza viruses of different origins, the impact of influenza virus infection on turkey gut and respiratory microbiota has not been demonstrated. In this study, we investigated the relationships between low pathogenicity avian influenza (LPAI) virus replication, cytokine gene expression, and respiratory and gut microbiota disruption in specific-pathogen-free turkeys. Differential replication of two LPAI H5N2 viruses paralleled the levels of clinical signs and cytokine gene expression. During active virus shedding, there was significant increase of ileal and nasal bacterial contents, which inversely corresponded with bacterial species diversity. Spearman’s correlation tests between bacterial abundance and local viral titers revealed that LPAI virus-induced dysbiosis was strongest in the nasal cavity followed by trachea, and weakest in the gut. Significant correlations were also observed between cytokine gene expression levels and relative abundances of several bacteria in tracheas of infected turkeys. For example, interferon γ/λ and interleukin-6 gene expression levels were correlated positively with *Staphylococcus* and *Pseudomonas* abundances, and negatively with *Lactobacillus* abundance. Overall, our data suggest a potential relationship where bacterial community diversity and enrichment or depletion of several bacterial genera in the gut and respiratory tract are dependent on the level of LPAI virus replication. Further work is needed to establish whether respiratory and enteric dysbiosis in LPAI virus-infected turkeys is a result of host immunological responses or other causes such as changes in nutritional uptake.

## Introduction

Influenza A viruses are highly contagious pathogens that continue to evolve in many avian and mammalian species causing threats to both animal and human health (Palese & Shaw, 2007). Many avian influenza viruses, including highly pathogenic avian influenza (HPAI) and low pathogenicity avian influenza (LPAI) viruses of H5, H7, and H9 subtypes, have the potential to transmit directly from poultry to human (Yassine et al., 2010). As an important poultry disease, avian influenza is associated with negative impacts on the global economy (Swayne & Suarez, 2000).

LPAI viruses are progenitors of all HPAI viruses. The LPAI viruses are naturally found in wild aquatic bird reservoirs and are occasionally transmitted to domestic poultry where they undergo mutation to HPAI phenotype (Yoon, Webby & Webster, 2014). Turkeys are highly susceptible to HPAI and LPAI viruses of different origins including wild birds, domestic birds, swine, and humans (Morales et al., 2009; Pantin-Jackwood et al., 2017; Pillai et al., 2010b; Sjurseth et al., 2017; Slomka et al., 2018) and, therefore, can promote generation of novel reassortant viruses of unknown pathogenicity. Furthermore, commercial turkey flocks infected with LPAI viruses often show increased mortalities, clinical signs such as respiratory distress and sinusitis, and poor production performance (Jimenez-Bluhm et al., 2019; Kleven, Nelson & Deshmukh, 1970; Reid et al., 2016; Zanella, Dall’Ara & Martino, 2001). Several controlled studies have demonstrated that the infectivity, transmissibility, and pathogenicity of LPAI virus in turkeys are strain dependent, with some strains being more virulent than others (Laudert et al., 1993; Morales et al., 2009; Pantin-Jackwood et al., 2017; Slomka et al., 2018; Spackman et al., 2010).

Avian influenza viruses replicate primarily in the epithelial cells of respiratory and gut mucosae that are colonized with commensal microbiota (Johnson et al., 2018; Ngunjiri et al., 2019b; Taylor et al., 2020). The gut microbiota of poultry species has been extensively studied due to its impacts on health and production performance metrics (Celi et al., 2017; Choi, Lee & Sul, 2015; Danzeisen et al., 2013; Jankowski et al., 2013; Johnson et al., 2018; Kurtoglu et al., 2004; Lei et al., 2013; Nava et al., 2005; Simon et al., 2016; Stanley et al., 2012; Torok et al., 2011). This has not been the case for respiratory microbiota and their contribution in health and production performance remains largely unknown. Our recent studies have provided novel insights into the dynamics of microbiota in respiratory tracts of commercial turkeys and chicken layers throughout the flock cycle (Ngunjiri et al., 2019b; Taylor et al., 2020) and across several successive broiler flock cycles within a vertically integrated system (Johnson et al., 2018). Respiratory bacteria in turkeys and chickens are mostly unique from gut bacteria, yet there is significant exchange of bacteria (including potential pathogens) between the respiratory and digestive tracts (Johnson et al., 2018; Ngunjiri et al., 2019b; Taylor et al., 2020). Further, dysbiosis (shift in the composition) of both gut and respiratory microbiota was observed, where increase or decrease of numerous bacterial taxa was associated with poor weight gain trajectories in chicken broilers and tom turkeys (Danzeisen et al., 2013; Johnson et al., 2018; Taylor et al., 2020). Such dysbiosis can result from physiological or immunological mediators induced by factors such as heat stress (Mashaly et al., 2004; Rostagno, 2020), type of nutrition (Emadi et al., 2011; Kidd, 2004; Swaggerty et al., 2019), and pathogen infection (Borges et al., 2018; Deriu et al., 2016; Ganz et al., 2017; Hird et al., 2018; Li et al., 2018; Wen et al., 2018; Yitbarek et al., 2018c; Zhao et al., 2018).

Upon infection by a pathogen, the host quickly responds by mounting broad spectrum antimicrobial innate immune responses such as production of interferons (IFNs), pro-inflammatory cytokines, and chemokines (Akira, Uematsu & Takeuchi, 2006; Medzhitov & Janeway Jr, 2000; Mogensen, 2009), which work in concert to neutralize the pathogen (Akira, Uematsu & Takeuchi, 2006; Medzhitov & Janeway Jr, 2000; Mogensen, 2009) and promote the development of pathogen-specific adaptive immunity (Clark & Kupper, 2005; Crampton, Voynova & Bolland, 2010; Cuddapah, Barski & Zhao, 2010; Hoebe, Janssen & Beutler, 2004; Le Bon & Tough, 2002). Induction of cytokines and other immune mediators by influenza viruses has been studied in chickens and Anseriformes birds (Cornelissen et al., 2013; Evseev & Magor, 2019; Gao et al., 2017; Guan, Fu & Sharif, 2015; Volmer et al., 2011). However, the nature and consequences of influenza virus-induced innate immune responses in turkeys has not been investigated.

Avian influenza viruses are among the numerous pathogens that induce strong innate immune responses such as IFNs and pro-inflammatory cytokines (Akira, Uematsu & Takeuchi, 2006; Medzhitov & Janeway Jr, 2000; Mogensen, 2009) which can trigger dysbiosis in the respiratory tract of mammals (Duvigneau et al., 2016; Engelsoy, Rangel & Demirel, 2019; Madouri et al., 2018; Sharma-Chawla et al., 2019) and in the avian gut (Ganz et al., 2017; Hird et al., 2018; Li et al., 2018; Yitbarek et al., 2018c; Zhao et al., 2018). However, influenza virus-induced dysbiosis of avian respiratory microbiota has not been described due to challenges in respiratory sampling methodologies (Abundo et al., 2020; Abundo et al., 2021). Furthermore, there is a lack of information about the impacts of influenza virus infection on turkey microbiota despite turkeys generally being highly susceptible to diverse influenza virus strains (Pantin-Jackwood et al., 2017; Pillai et al., 2010b; Tumpey, Kapczynski & Swayne, 2004). In this study, we investigated the relationships between LPAI virus replication, cytokine gene expression, and respiratory and gut microbiota disruption in 18-day old specific-pathogen-free turkeys. The study demonstrates a potential causal relationship where bacterial community diversity and enrichment or depletion of several bacterial genera in the gut and respiratory tract are dependent on the level of LPAI virus replication. Furthermore, LPAI virus infection induced the expression of host cytokine genes in trachea, which strongly correlated with bacteria enrichment or depletion.

## Materials & Methods

### Turkeys and ethics statement

Specific pathogen free (SPF) turkeys were obtained from the Food Animal Health Research Program (OARDC, OSU) flock (Ngunjiri et al., 2019a) and maintained free from known turkey pathogens including reovirus, avian encephalomyelitis virus, infectious bursal disease virus, infectious bronchitis virus, Newcastle disease virus, avian influenza virus, hemorrhagic enteritis virus, *Mycoplasma gallisepticum*, *M. synoviae*, *M. meleagridis*, *Bordetella avium* and *Salmonella pullorum*. The turkeys were housed and acclimatized in a BSL-2 facility with forced air ventilation and adequate air exchanges to prevent ammonia build up. Air entering or leaving the facility is HEPA filtered. At 2 weeks of age, birds were randomly picked from the brooders, assigned to one of the experimental groups, tagged, transferred to isolators (Model 934–1, 900 sq. inch, Federal Designs Inc., Comer, GA) inside the BSL-2 facility, and experiments were conducted in accordance with protocol #2009AG0002-R2 approved by The Ohio State University Institutional Animal Care and Use Committee (IACUC). Room and isolator temperatures were maintained at 25 ± 3 °C. The animals were provided with *ad libitum* access to feed and water and their welfare monitored twice daily. They were humanely euthanized at 5 (1st sampling time point) or 14 days post-infection (2nd sampling time-point and experiment endpoint), or when they displayed symptoms such as ruffled feathers and reluctance to move, not moving when prodded, respiratory distress, or injuries that were not related to experimental treatment. Administration of analgesia drugs was unnecessary since the birds were euthanized soon after the emergence of clinical symptoms. Humane euthanasia was actualized by exposure to carbon dioxide (CO_2_) as previously described (Elaish et al., 2017; Elaish et al., 2019; Ghorbani et al., 2019; Jang et al., 2018). Three poults were placed in the euthanasia chamber connected to a CO_2_ source, with CO_2_ flow set at 10–30% displacement of chamber volume/minute. Birds were observed for respiratory arrest and lack of heartbeat and the CO_2_ flow was maintained for at least one minute after the respiratory arrest and lack of heartbeat were observed. Upon observation of death, an additional secondary physical euthanasia (cervical dislocation or removal of a vital organ) was performed before collection of tissues and carcass disposal (Elaish et al., 2017; Elaish et al., 2019; Ghorbani et al., 2019; Jang et al., 2018).

### Experimental design

A total of 96 poults were placed in six isolators at 10 days of age. Each bird was randomly picked from the brooder and assigned to one of isolators. The first bird was assigned to Isolator #1, second bird to isolator #2, and so on, and the sequence was repeated until 16 birds were placed in each isolator. After an 8-day acclimatization period, the birds were assigned into three treatment groups (2 isolators/group; 32 birds/group) and inoculated with PBS-only (“Mock”) or H5N2 low-pathogenicity avian influenza viruses: CK/PA/13609/93 (CKPA) and TK/MN/10734-2/95 (TKMN). The treatment group was the experimental unit. Sixteen birds per group were sufficient for statistical comparisons of viral shedding, gene expression, and microbiota data based on our published studies (Ghorbani et al., 2020; Jang et al., 2018; Johnson et al., 2018; Ngunjiri et al., 2019b; Taylor et al., 2020). Median egg infectious dose (EID_50_) for each virus stock was calculated prior to normalization of virus concentration on the day of challenge. Each bird was intrachoanally inoculated with 200 µL of PBS or virus (10^6^ EID_50_). At 5 days post infection (dpi), 8 birds from each isolator were euthanized for necropsy. At 14 dpi, the remaining birds were necropsied. Five birds in the CKPA-treated group developed severe clinical signs and were euthanized at 4 dpi and between 6 and 7 dpi. These birds were not sampled for microbiota because our target sampling time-points were at 5 and 14 dpi. Animal handling procedures were done starting from the Mock group, then the infected groups to reduce confounders. All personnel involved in this study were aware of group allocation of the animals. The research question, experimental design, and data analysis protocols were not registered before the study.

### Quantification of hemagglutination inhibition (HI) antibodies

To determine homologous serum HI antibody titers, blood was drawn from live birds via the brachial (wing) vein for serum collection prior to euthanasia at 14 dpi. The blood was kept overnight at room temperature for serum separation to occur. The separated sera were decanted into two mL tubes, inactivated at 60 °C for 30 min, and stored at −20 °C until used for HI test. The HI test was conducted as described previously (Jang et al., 2018). In brief: 50 µl of heat-inactivated serum was serially diluted in 96-well plates and mixed with an equal volume of homologous virus preparation containing eight hemagglutinating units of the virus, the serum-virus mixture was incubated for 30 min at room temperature, and 50 µl of 1% turkey erythrocyte suspension was added in each well and mixed with the virus-antibody complexes using a benchtop shaker. The HI titer was determined as the reciprocal of end-point dilution showing complete inhibition of hemagglutination.

### Transcriptional analysis

Transcriptional analysis of several innate immune genes was performed with trachea samples collected during necropsy at 5 dpi, as described in our previous studies (Ghorbani et al., 2020; Jang et al., 2018; Jang, Ngunjiri & Lee, 2016; KC et al., 2020). The uppermost 0.5 cm of tracheal tissue was excised using sterile scissors and forceps, placed into a tube with one mL TRIzol, snap-frozen in liquid nitrogen, and stored at −70 C until processing. Later, 0.6 mL dry volume mixed 0.5 mm and 0.1 mm ZR BashingBead lysis matrix (Zymo Research, Tustin, CA, USA) was decanted into tubes containing the tracheal samples. The tissues were then homogenized using a TissueLyser II (Qiagen Sciences Inc., Germantown, MD) at a frequency of 30 Hz for 5–7 min. Once the tissue was completely homogenized, chloroform was added to the TRIzol at a ratio of 1-part chloroform to 5-parts TRIzol. The mixture was vortexed vigorously for 15 s and centrifuged (16,000× g, 4 °C, 20 min). Crude RNA (the top, transparent layer) was transferred to a new tube, taking care not to disturb the white DNA layer below. An equal volume of EtOH was added to the tube and the mixture was vortexed before being transferred in entirety to a Zymo-Spin IIC Column (Zymo Research, Tustin, CA, USA) and centrifuged (10,000× g, 30 s). 400 µL RNA Wash Buffer were added to the column, which was centrifuged (16,000× g, 30 s), transferred to a new collection tube, and centrifuged again (16,000× g, 2 min) to ensure that the filter was dry. Further steps in the Direct-zol RNA MiniPrep Plus Quick Protocol (Zymo Research, Tustin, CA, USA) were followed, including the recommended DNase I treatment step. Centrifugation for Steps 3–4 was conducted at 16,000× g for 30 s. And additional centrifugation (to ensure filter dryness) was added after Step 4 (16,000× g, 2 min). Final elution volume was changed to 50 µL, incubation time was increased to 1 min (room temperature), and final centrifugation was conducted at 10,000× g for 1 min. The RNA was stored at −80 °C until use.

Complementary DNA (cDNA) was synthesized using GoScript reverse transcriptase (Promega, Madison, WI, USA) with oligo(dT)15 primer. RNA samples were thawed and quantified using NanoDrop™ 2000 (Thermo Fisher Scientific, Waltham, MA, USA) and diluted to a standard concentration of 250 ng per 10 µL aliquot before adding the 2 µL of Oligo(dT)_15_ primer (pre-diluted to 250 ng/ µL). The RNA-primer mixture was incubated at 72 °C for 5 min and immediately placed on ice. Next, 8 µl of master mix containing 4 µl of GoScript Reaction Buffer (5X), 2 µl of MgCl2 (25 mM), 1 µl of deoxynucleoside triphosphate (dNTP) mix (containing 10 mM of each dNTP), and 1 µl of reverse transcriptase enzyme (160 U/ µL) was added to each tube and synthesis of the first cDNA strand was allowed to take place at 42 °C for 1 h. Then, quantitative PCR (qPCR) was performed using PerfeCTa SYBR green FastMix Low ROX kit (QuantaBio, Beverly, MA, USA) in a 7500 real-time PCR system (Applied Biosystems, Waltham, MA, USA). Briefly, cDNA was diluted 1.5625 times and 5 µl of the diluted cDNA was mixed with 15 µl of master mix containing 10 µl of SYBR Green FastMix (2X) and 2.5 µl of each primer (20 µM) in a 0.2-ml tube. The cycle conditions of quantitative PCR were 95 °C for 10 min, followed by 40 cycles of 95 °C for 15 s and 60 °C for 1 min. Afterward, melting curve analysis was performed from 60 °C to 90 °C. Primers for amplification of IFN-α, IFN-*β*, IFN-γ, interleukin-6 (IL-6), and lipopolysaccharide-induced tumor necrosis factor-*α* factor (LITAF) genes were published in an earlier study (Sharafeldin et al., 2015), while those for amplification of 2′–5′-oligoadenylate synthase (OAS) and myxovirus (influenza virus) resistance 1 (Mx), IFN-*λ*, and glyceraldehyde-3-phosphate dehydrogenase (GAPDH) genes were designed in this study. All primer sequences, accession numbers for the target genes, and sizes of the amplified products are provided in Table 1. Differential gene expressions were normalized using GAPDH gene as the internal control. Relative mRNA expression levels were calculated as fold changes over the mock group using the 2−ΔΔCT method (Ghorbani et al., 2020; Jang et al., 2018).

**Table 1:** List of target genes, primer sequences, and RT-PCR product sizes.

Target gene	GenBank accession #	Primer sequence	Product size
IFN-α	U28140.1	F 5′GCCTCCTCAACCAGATCCAG 3′	108 bp
		R 5′TGATGGTGAGGTGAGGGTTG 3′	
IFN-β	XM_003213368.1	F 5′CCGTTCTGGAAAGCAAGGAC 3′	119 bp
		R 5′GTGTGCGTGGTCAATCCAGT 3′	
IFN-γ	AJ000725	F 5′TGAAGGCAGTGCGGAGGATG 3′	115 bp
		R5′AGGGTGATCCGGTCAGGT 3′	
IFN-λ	XM_003207378.3	F5′TGAAGGCAGTGCGGAGGATG 3′	130 bp
		R5′AGGGTGATCCGGTCAGGT 3′	
IL-6	XM_003207130.1	F 5′GCTTCGACGAGGAGAAATGC 3′	120 bp
		R 5′AGCACAGCGATTCGACATTC 3′	
LITAF	XM_003210543.1	F5′TGACTTGGCTGTCGTGTGGT 3′	119 bp
		R 5′GGCATTGCAATTTGGACAGA 3′	
OAS	XM_019619951.2	F5′ CATGGCCTCTTCTACAATG 3′	112 bp
		R5′TGGGCCATACGGTGTAGACG 3′	
Mx	XM_003202961.4	F5′GCTGCCAAGGCCAGAATTG 3′	139 bp
		R5′CCCTGGCAGCTTTTAAATCATC 3′	
GAPDH	NM_001303179.1	F5′CTGGCAAAGTCCAAGTGGTG 3′	123 bp
		R5′TCCCATTCTCAGCCTTGACA 3′	

### Viral titration by quantitative real-time RT-PCR

Virus quantification was performed using supernatants from gut sample (cecum and ileum tissues and digesta contained therein) homogenates and respiratory tract (nasal and tracheal) washes. Procedures for respiratory washes and gut sample are detailed in our recent reports (Abundo et al., 2020; Ngunjiri et al., 2019b; Taylor et al., 2020). Gut sample supernatants were generated by adding 500 µL of sterile PBS to tubes containing 0.3 g of homogenates followed by vigorous vortexing for 2 min and centrifugation at 10,000× g at 4 °C for 5 min. Respiratory sample supernatants were generated by centrifugation of tracheal and sinus washes at 10,000× g at 4 °C for 10 min. Viral RNA (vRNA) was extracted from 200 µL of supernatant using RNeasy Mini Kit (Qiagen Sciences Inc., Germantown, MD, USA). The amount of viral genomes was determined using quantitative reverse transcription PCR (qRT-PCR) with type A influenza virus matrix gene specific primers and probes, and EID_50_ viral titers were extrapolated from standard curves generated with the stocks used for infection as described in our previous reports (Ghorbani et al., 2019; Jang, Ngunjiri & Lee, 2016). To generate a standard curve, viral RNA was extracted from serial 10-fold dilutions of the same virus stock (with known EID_50_ titer) used to inoculate the turkeys and subjected to qRT-PCR to generate cycle threshold (Ct) values. A scatter plot of Ct values (*x*-axis) against log_10_-transformed virus dilution factors (*y*-axis) was made and fitted with a linear trendline to generate the equation *y* =*bx + a*, where *y* is the virus concentration (log_10_ EID_50_/ml), *b* is the slope of the trendline, *x* is the Ct value, and *a* is the y-intercept. This equation was then used to extrapolate gut and respiratory tract viral titers by substituting the Ct values obtained from sample-derived viral RNA for *x*.

### Bursal atrophy

Bursal atrophy was determined based on bursa-body weight ratio (Cazaban et al., 2015). Body weight of individual birds were taken immediately after euthanasia before any tissues were excised. Full-size bursas were carefully excised during necropsy and weighed individually. The bursa-body weight ratio was calculated as follows: bursa-body weight ratio = [bursa weight (g)/body weight (g)] ×1000 (Stoute et al., 2013).

### Sample collection and processing for 16S rRNA gene sequencing

Samples were collected from euthanized birds at 5 and 14 dpi. Collection, storage, and processing of respiratory and gut samples, including sinus and tracheal washes and gut (ileum and cecum) sample homogenization, were performed exactly as detailed in our recent reports (Abundo et al., 2020; Ngunjiri et al., 2019b; Taylor et al., 2020). For lower respiratory tract lavage collection, we assembled an apparatus by fitting a sterile non-filtered 200 µL pipet tip to a sterile 25 mL Luer Lock syringe. The bottom 3–5 mm of the tip was then trimmed with sterile scissors to broaden the channel. The syringe was filled with of sterile PBS before inserting the pipet tip into the lower trachea (about 0.5 cm above the syrinx) and securing it airtight with forceps before pumping the PBS into the lower respiratory tract. This process was repeated until about 40 mL of PBS filled the lower respiratory tract. After the last volume of PBS was added, the PBS was flushed in and out of the lower respiratory tract several (∼15–20) times until it became cloudy. As much of the PBS was retrieved as possible (typically yielding ∼30 mL) and transferred to a sterile tube. The tubes were kept on ice until processing. Later, the tubes were centrifuged at 7,830 ×g for 20 min. The supernatant was decanted, and pellets transferred to two mL for storage at −70 °C until DNA extraction.

### DNA extraction for 16S rRNA gene analysis

DNA was extracted from respiratory wash pellets using the DNeasy Blood & Tissue kit (Qiagen Sciences Inc., Germantown, MD, USA) as described previously (Ngunjiri et al., 2019b) with some specific modifications, which were optimized based on sample type to improve DNA yield and minimize PCR inhibitors. In particular, enzymatic lysis buffer was used instead of Buffer ATL. Lysis buffer and reconstituted lysozyme were prepared beforehand and stored separately. Lysozyme was added to lysis buffer immediately before use at concentrations of 0.02 M for nasal wash samples, and 0.031M for tracheal and lower respiratory lavage samples. The disparity in reagent concentrations is due to the volumes of PBS added to samples to resuspend pellets.

Lysozyme was reconstituted by slowly adding approximately 1.75 mL sodium acetate (C_2_H_3_NaO_2_) to 1.5 g lysozyme desiccate in a 15mL round-bottom tube and vortexing until thoroughly dissolved. Once the mixture was suspended, three mL of sterile glycerol was added and vortexed. The final volume was six mL, with a concentration of 250 mg/mL, was aliquoted to two mL screwcap tubes (250 µL/tube) and stored at −20 °C until use.

For nasal wash pellets, lysis buffer was prepared by combining Tris reagent (pH 8), EDTA, and Triton X to final concentrations of 0.02 M, 0.002 M, and 1.2%, respectively, in nuclease-free water. Lysis buffer for tracheal wash and lower respiratory lavage pellets was prepared in a similar manner except that the final concentrations were 0.031 M, 0.0031 M, and 1.867%, respectively. Lysis buffer was stored at room temperature until use.

Nasal wash pellets were resuspended in 180 uL of enzymatic lysis buffer (lysis buffer plus lysozyme at 20 mg/ml) by adding 2 sterile glass beads and vortexing to break up the pellet. Samples were incubated at 37 °C for 2 h. After adding 25 µL of proteinase K and 200uL Buffer AL, samples were incubated again at 56 °C for 30 min. A 200 µL volume of 100% ethanol was added to each sample, and the total sample volume was transferred to a filter spin column and centrifuged (10,000×g for 1 min). The column was washed by applying 730 µL of Buffers AW1 and AW2, with centrifuging between each wash (10,000×g, 1 min). To elute DNA from the column filters, 50 µL of Buffer AE was applied directly to the center of the column, then the samples were incubated at room temperature for 5 min and centrifuged (10,000×g, 1 min). The eluate was reapplied to the filter, incubated, centrifuged a second time.

The procedure for tracheal wash and lower respiratory lavage pellets differed from the above protocol for nasal wash samples in the following ways. First, 500 µL of sterile 1X PBS was used to resuspend the pellet, then 900 uL enzymatic lysis buffer was added. Second, one mL of Buffer AL was added after the first incubation step (37 °C for 45 min), and one mL 100% ethanol was added after the second incubation step (56 °C for 30 min). Running the total volume of solution through the spin column required 5-6 rounds of centrifugation. Finally, 500 µL of Buffers AW1 and AW2 were used to wash the filter.

The protocol for DNA extraction from ileum and cecum homogenate samples was modified from the DNeasy PowerSoil Kit and the extraction was performed exactly as detailed in our recent report (Ngunjiri et al., 2019b).

### 16S rRNA gene amplification, quantitation, and sequencing

Our optimized methods for sequencing and sequence processing are detailed in our recent reports (Abundo et al., 2021; Taylor et al., 2020). For each sample, DNA was quantified using NanoDrop 2000c spectrophotometer (Thermo Fisher Scientific, Waltham, MA, USA), adjusted to a concentration of 100 ng/µL, and shipped to the University of Minnesota Genomics Center (Minneapolis, MN, USA) for sequencing. The number of 16S rRNA gene copies in each sample was determined through quantitative PCR (qPCR) of the V4 hypervariable region using primer sets 515F (5′-TCGTCGGCAGCGTCAGATGTGTATAAGAGACAGGTGCCAGCMGCCGCGGTAA-3′) and 806R (5′-GTCTCGTGGGCTCGGAGATGTGTATAAGAGACAGGGACTACHVGGGTWTCTAAT-3′) Nextera primers (Caporaso et al., 2012). The conditions for qPCR were as follows: one cycle of 95 °C for 5 min; followed by 35 cycles of 98 °C for 20 s, 55 °C for 15 s, and 72 °C for 1 min; and one cycle of 72 °C for 5 min using QuantStudio Real-Time PCR System (Thermo Fisher Scientific, Waltham, MA, USA). The Cycle Threshold (CT) values were then converted to 16S rRNA gene copy numbers using a standard curve. The number of copies per ng of DNA was estimated by the ratio of total 16S rRNA copies to the DNA concentration/uL multiplied by the volume of DNA used in the copy quantification step (5 µL) and used as a proxy for bacteria content. Group comparisons for the bacterial content were performed using the Wilcoxon rank sum test with Holm correction (‘stats::pairwise.wilcox.test’).

Before sequencing, the samples were normalized to 1.67 × 10^5^ 16S rRNA gene copies/ µL then subjected to two rounds of PCR. In the first round, the 515F and 806R Nextera primers were used for the amplification of V4 region from 3 *μ*L of normalized DNA using the following cycling parameters: one cycle of 95 °C for 5 min, followed by 25 cycles of 98 °C for 20 s, 55 °C for 15 s, and 72 °C for 1 min. The PCR product was then diluted to 1:100, and 5 µL was used in the second round of PCR using forward (5′-AATGATACGGCGACCACCGAGATCTACAC(i5)TCGTCGGCAGCGTC–3′) and reverse (5′-CAAGCAGAAGACGGCATACGAGAT(i7)GTCTCGTGGGCTCGG–3′) indexing primers (Integrated DNA Technologies, Coralville, IA). In the second PCR, the following cycling parameters were used: 1 cycle at 95 °C for 5 min, followed by 10 cycles of 98 °C for 20 s, 55 °C for 15 s, and 72 °C for 1 min. Pooled size-selected samples were denatured using NaOH, diluted to 8 pM in Illumina’s HT1 buffer, spiked with 20% PhiX, and heat denatured at 96 °C for 2 min immediately before loading. MiSeq 600 (2 × 300 bp) cycle v3 kit (Illumina, San Diego, CA, USA) was used to sequence the samples. This protocol is an optimization of the Earth Microbiome Project protocol (Gilbert & Meyer, 2012; Gohl et al., 2016).

The barcoded Illumina sequencing data files were demultiplexed to generate fastq files for each sample followed by merging of R1 and R2 reads for each sample, removal of the amplification and Kit adapter primer sequences, and removal of very short (<245 bp) and very long (>260 bp) sequences.

The merged sequences were imported into QIIME2 (qiime2-2019.1) (Bolyen et al., 2019) for denoising through removal of PCR chimeras, clustering of amplicon sequence variants (ASVs) with 100% identities, removal of non-16S rRNA gene ASVs, generation of a feature table with ASV occurrence frequencies, generation of a mid-rooted phylogenetic tree, and taxonomic classification using SILVA 16S rRNA gene database (release 132). The elaborate protocol including all software used is described in our recent report (Taylor et al., 2020).

### 16S rRNA gene sequence analysis

Using QIIME2 (qiime2-2019.1) (Bolyen et al., 2019), the feature table was rarefied to 5,000 reads per sample by calling the ‘core-metrics-phylogenetic’ command in the ‘diversity’ plugin. All microbiome analyses reflect the numbers of samples/group given in Table 2. All other analyses were performed in RStudio (R Core Team, 2019) using the indicated packages. Alpha diversity was quantified as the number of unique ASVs observed per sample (‘vegan::specnumber’) (Oksanen et al., 2013). Group comparison was performed using pairwise Wilcoxon rank sum tests with Holm correction (‘stats::pairwise.wilcox.test’). Beta diversity was quantified as the total abundance-weighted phylogenetic distance between the constituent ASVs of one sample and another (‘GUniFrac::GUniFrac’) (Chen, 2018). Group comparisons were performed using pairwise PERMANOVA with Holm correction (‘pairwiseAdonis::pairwise.adonis’) (Martinez Arbizu, 2017).

Predominant taxa were identified for each body site as those ASVs that were present in all birds of at least one treatment group at a mean relative abundance of >1%. In order to draw more general conclusions from these taxa, the definition was expanded to encompass all ASVs belonging to the same lowest taxonomic classification (assigned by SILVA) as the identified predominant taxa. The site, infection group, and number of ASVs for each identified taxon is described in Table S1.

Differential abundance analysis was performed on this taxonomic set by taking the ratio of mean relative abundance (percent) of a genus for each virus-treated group to the mean relative abundance of the Mock group. The second log (Log_2_) of this value describes the fold change of mean abundance in the virus-infected groups from the Mock. Statistical comparisons were performed using the Wilcoxon rank-sum test on untransformed values. *P*-values were adjusted post-hoc using the Holm method (‘stats::p.adjust’).

**Table 2:** Number of samples per group after rarefaction to 5,000 reads.

		**5 dpi**				**14 dpi**		
Body site		Mock	TKMN	CKPA		Mock	TKMN	CKPA
NAS		16	16	16		16	16	11
TRA		16	16	16		15	15	5
LRT		16	16	16		16	15	10
CEC		16	16	16		16	16	11
ILE		13	16	16		5	15	10

The distribution of predominant taxa among body sites and treatments was profiled according to presence at a treatment-level consensus relative abundance of >0.5%. The distribution of the taxa thus defined was necessary to ensure that correlations were performed with sufficient non-zero data. Relative abundance data were used for Spearman correlation (‘stats::cor’) with local viral titers (in nasal cavity, trachea, cecum, and ileum) and with local gene expression (trachea only). Significance of the correlation coefficient was assigned from a pairwise *t*-test comparison built into ‘psych::corr.test’ (Revelle, 2019), adjusted with the Holm method.

### Sequence data and software availability

Raw data files and metadata are publicly available in the NCBI BioProject database under accession number PRJNA644054. Raw data files are also available through the Sequence Read Archive (SRA) under accession numbers SAMN15446589– SAMN15447100. Pipe scripts for QIIME2 sequence processing and generation of the ASV table and R scripts for analysis are publicly available at https://github.com/kjmtaylor22/cwl-cats.

## Results

### Differential viral replication parallels the levels of survival, immune-related gene expression, bursal atrophy, and serum hemagglutination inhibition antibodies

**Figure 1:**
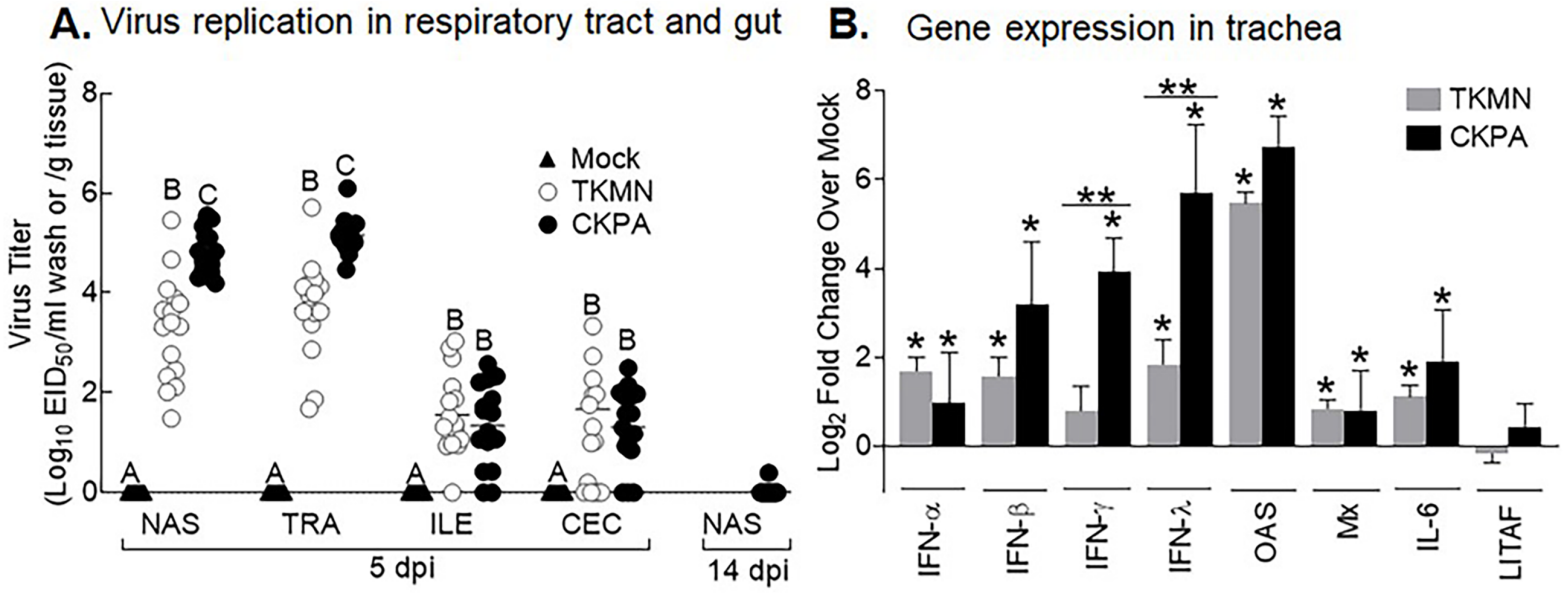
Virus replication and host gene expression. (A) Viral titers in washes from tracheas and nasal cavities and in supernatants of ileal and cecal homogenates were determined by qRT-PCR. Virus titers are expressed as median egg infectious doses per ml of wash or gram of tissue. Different letters inside the plot indicate statistical significance among groups (ANOVA, *p* < 0.05). (B) Gene expression levels at 5 days post-infection (dpi). The bars represent Log2 fold change in gene transcription level over mock-infected controls. Error bars represent mean ± SD (*n* = 16 birds per group at 5 dpi). Asterisks without horizontal bar indicate significant differences compared with the Mock group. Horizontal bar with asterisks indicates significant difference between virus-infected groups (Kruskal-Wallis test, * *p* < 0.05, ** *p* < 0.01). CKPA, infected with A/CK/PA/13609/93 (H5N2) virus. TKMN, infected with A/TK/MN/10734-2/95 (H5N2) virus. NAS = nasal cavity, TRA = trachea, LRT = lower respiratory tract, CEC = cecum, ILE = ileum. IFN = interferon. OAS = 2′–5′-oligoadenylate synthase, Mx = myxovirus (influenza virus) resistance 1, IL-6 = interleukin-6, LITAF = lipopolysaccharide-induced tumor necrosis factor-α factor.

To enable differential assessment of quantitative and qualitative differences in microbiota between virus infected and mock infected poults, we chose two H5N2 viruses (CKPA, isolated in chicken, and TKMN, isolated in turkey) that replicate at different levels in turkeys (Pillai et al., 2010a). In accordance with our previous observation (Pillai et al., 2010a), five poults in the CKPA group developed severe clinical signs such as gasping and swollen heads and were euthanized at 4, 6, and 7 days-post-infection (dpi). These birds were not sampled because they were inadequate for statistical tests and the experimental design specifically called for quantitative analysis of microbiota at 5 and 14 dpi and tracheal gene expression at 5 dpi. Birds in the Mock and TKMN groups did not exhibit clinical signs throughout the duration of the experiment (14 days). At 5 dpi, poults in the CKPA group shed significantly higher viral titers in the upper respiratory tract (URT) (nasal cavity and trachea) than the TKMN group (Fig. 1A). Viral titers in the lower intestinal tract (LIT) (ileum and cecum) were significantly lower than URT and statistically indistinguishable between CKPA and TKMN groups. Viral clearance in the infected birds was confirmed with nasal wash samples collected from the CKPA group at 14 dpi (Fig. 1A). Accordingly, TKMN virus was assumed to be cleared since its titers tended to be equal or lower than CKPA virus. No virus was detected in gut or respiratory samples from the Mock group.

**Figure 2:**
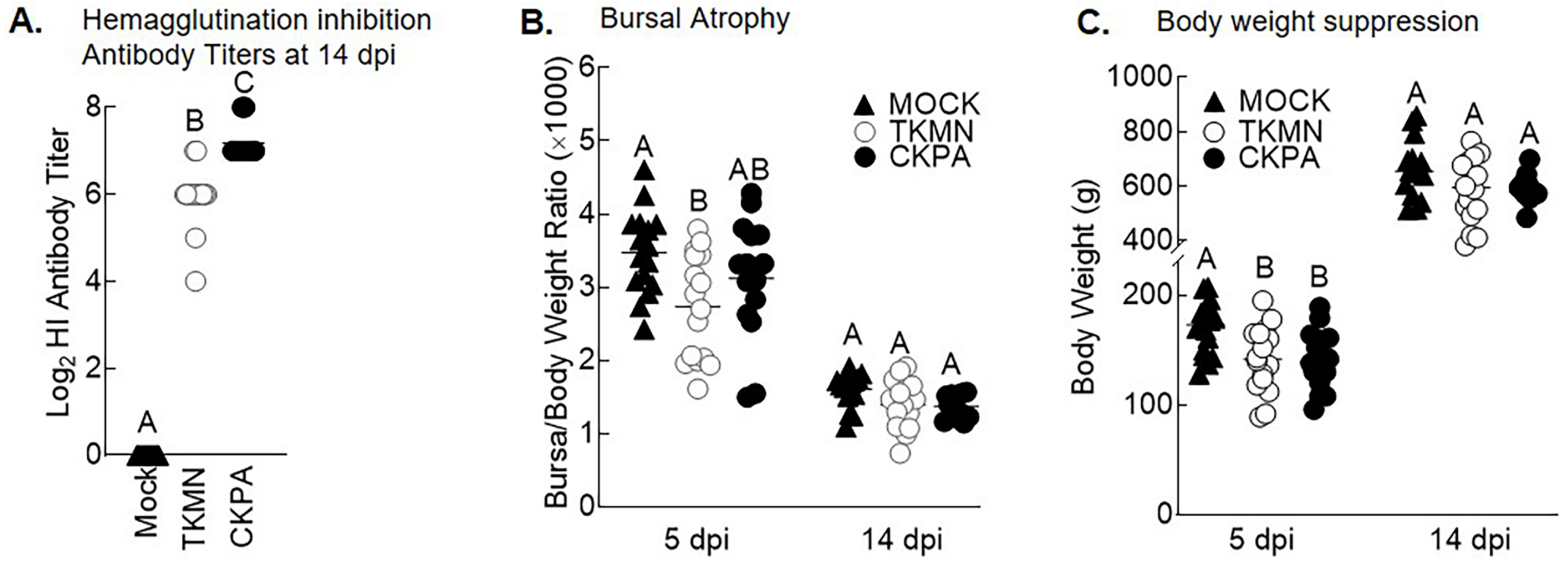
Serum antibody responses, bursal atrophy, and body weights. (A) Serum was collected at 14 days-post-infection (dpi) and tested for the presence of influenza virus A/CK/PA/13609/93 (CKPA; *n* = 10) and A/TK/MN/10734-2/95 (TKMN; *n* = 16) specific hemagglutination inhibition (HI) antibodies. (B) Bursal/body weight ratios at 5 and 14 dpi. (C) Body weights at 5 and 14 dpi. Different letters inside the plot indicate statistical significance among groups (ANOVA, *p* < 0.05). At 5 dpi, *n* = 16 birds/group. At 14 dpi, *n* = 16 birds in Mock and TKMN groups, and *n* = 10 birds in CKPA group.

LPAI virus infection results in local and systemic immune-related gene expression in avian species (Bergervoet et al., 2019; Xing et al., 2008). Here, we focused on local gene responses in trachea due to its tendency to shed higher titers of each virus than the other sites (Fig. 1A). The immune-related genes encompassed three categories: antiviral cytokines (IFN-α, IFN-β, IFN-γ, IFN-λ) (Schneider, Chevillotte & Rice, 2014), interferon inducible genes (OAS and Mx), and proinflammatory cytokines (IL-6, IFN-γ, LITAF) (Zhang & An, 2007). Except for lipopolysaccharide-induced tumor necrosis factor-α factor (LITAF), the genes examined here may be induced by double-stranded RNA produced as a replication intermediate by RNA viruses, including the influenza virus. Relative to the expression in mock-infected birds at 5 dpi, there was significant upregulation of IFN-α, IFN-β, IFN-λ, OAS, Mx, and IL-6 by both viruses, and IFN-γ by CKPA virus (Fig. 1B). It is worth noting that the levels of IFN-γ and IFN-λ mRNA induced by CKPA virus were about eightfold higher than those induced by the TKMN virus (*p* < 0.01). Only minor non-significant changes in the expression of LITAF were observed in virus-infected poults (Fig. 1B). Data from infected (CKPA and TKMN) and non-infected (Mock) birds were analyzed to demonstrate the relationship between virus replication and host gene expression through a series of regression plots (Fig. S1), with viral titer (Log_2_ EID_50_/ml) and gene expression level (Log_2_ fold change over Mock) as variables. For the CKPA group, the strength of correlation decreased in the following order: OAS (*r*^2^ = 0.94) >IFN-λ (*r*^2^ = 0.84) >IFN-γ (*r*^2^ = 0.71) >IFN-β (*r*^2^ = 0.53) >IL-6 (r^2^ = 0.51) >Mx (*r*^2^ = 0.27) >IFN-α (*r*^2^ = 0.25) >LITAF (*r*^2^ = 0.17). The order of the strength of correlation was different for TKMN virus: OAS (*r*^2^ = 0.87) >IFN-β (*r*^2^ = 0.43) = IFN-α (*r*^2^ = 0.43) >IFN-λ (*r*^2^ = 0.41) >IL-6 (*r*^2^ = 0.37) >Mx (*r*^2^ = 0.27) >IFN-γ (*r*^2^ = 0.22) >LITAF (*r*^2^ < 0.01). Noteworthy, the CKPA virus showed significantly higher serum HI antibody titers at 14 dpi compared to TKMN virus (Fig. 2A), while the TKMN group displayed significant bursa atrophy at 5 dpi compared to the Mock group, although a complete recovery was observed at 14 dpi (Fig. 2B). Additionally, LPAI virus infection resulted in significant suppression of body weight at 5 dpi, with TKMN and CKPA being statistically indistinguishable (Fig. 2C). However, no significant body weight suppression was observed at 14 dpi (Fig. 2C).

### Commensal microbiota is disrupted during active virus replication

**Figure 3:**
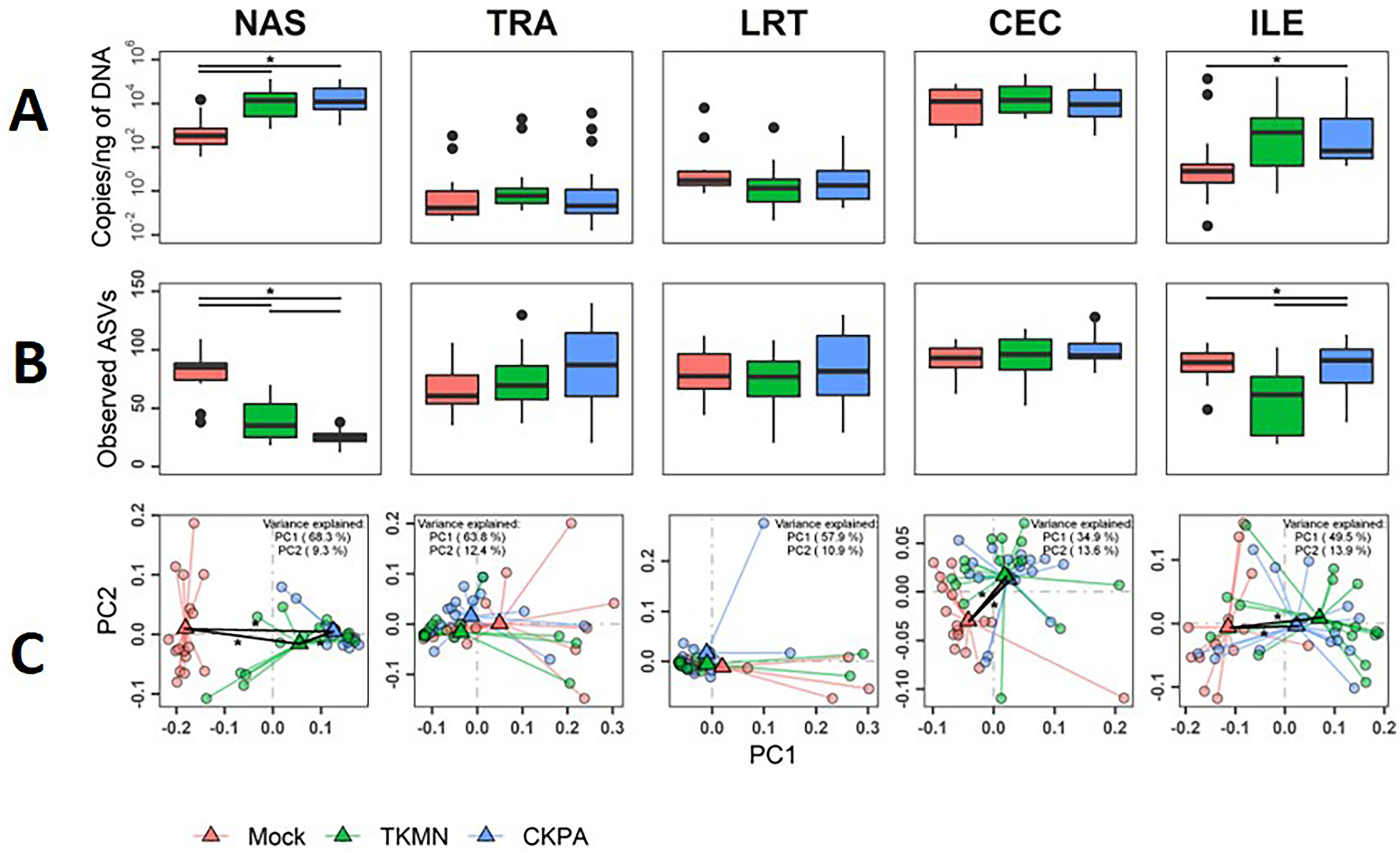
The density, diversity, and composition of the bacterial microbiota in the gut and respiratory tract at 5 days-post-infection. (A) Density was measured as the number of 16S rRNA gene copies present per nanogram (ng) of DNA used for sequencing. (B) Alpha diversity was measured as the number of distinct ASVs observed per sample after dataset rarefaction to 5,000 reads/sample. (C) Bacterial composition per sample was measured as the total abundance-weighted phylogenetic (UniFrac) distance between the bacteria of each sample. Black bars with stars indicate significant differences (*p* < 0.1) between groups. Significance in (A) and (B) observed with pairwise Wilcoxon rank sum test with Holm correction for multiple comparisons. Significance in (C) observed with pairwise PERMANOVA with Holm correction for multiple comparisons. See Table 1 for sample numbers/group. NAS = nasal cavity, TRA = trachea, LRT = lower respiratory tract, CEC = cecum, ILE = ileum.

Although commensal microbiota may demonstrate inhibitory effects against virus pathogens including influenza virus (Yitbarek et al., 2018a; Yitbarek et al., 2018b), the microbiota itself can be disrupted by virus-induced innate immune responses and pathologic effects. Here, we examined changes in bacterial microbiota during active virus replication (5 dpi), focusing on changes to total bacterial content (measured by 16S rRNA gene copies per nanogram of DNA per sample), diversity, composition, and abundance. The virus infected groups displayed significant increase in total bacterial content of the nasal cavity and ileum when compared to the Mock group, but not to each other (Fig. 3A). The increase in total bacterial content in the nasal cavity and ileum tended to be inversely associated with bacterial species richness (ASVs observed in each sample) (Fig. 3B). In the nasal cavity, although species richness was significantly decreased by both viruses compared to mock-infected birds, the average number of ASVs observed in the CKPA group was significantly lower than the TKMN group. In the ileum, only the TKMN group displayed a significant reduction of species richness compared to either the Mock or CKPA groups.

Dissimilarities in bacterial composition amongst birds in different treatment groups (beta diversity) were measured using abundance-weighted UniFrac distances. Following the trends observed in total bacterial content and alpha diversity (Figs. 3A and 3B), the ileum, cecum, and nasal cavity displayed significant virus-induced changes in beta diversity. The compositions of nasal microbiota in all three treatment groups were significantly different from one another (Fig. 3C, Table S2). For ileal and cecal microbiota, both virus-infected groups were significantly different from the Mock group, but not from each other.

To determine the impacts of virus infection on taxonomic composition of respiratory and gut microbiota, we targeted the taxa that were present in all birds of at least one treatment group at a mean relative abundance of >1%. These taxa were selected for each group and body site independently, and then summarized to genus level. Those taxa not classified to genus level were collapsed to family or order level while those identified as contaminants were dropped from further analysis, resulting in 23 taxa (Table S1). Contaminants were conservatively identified as ASVs present in all negative extraction control samples using the same extraction kit.

**Figure 4:**
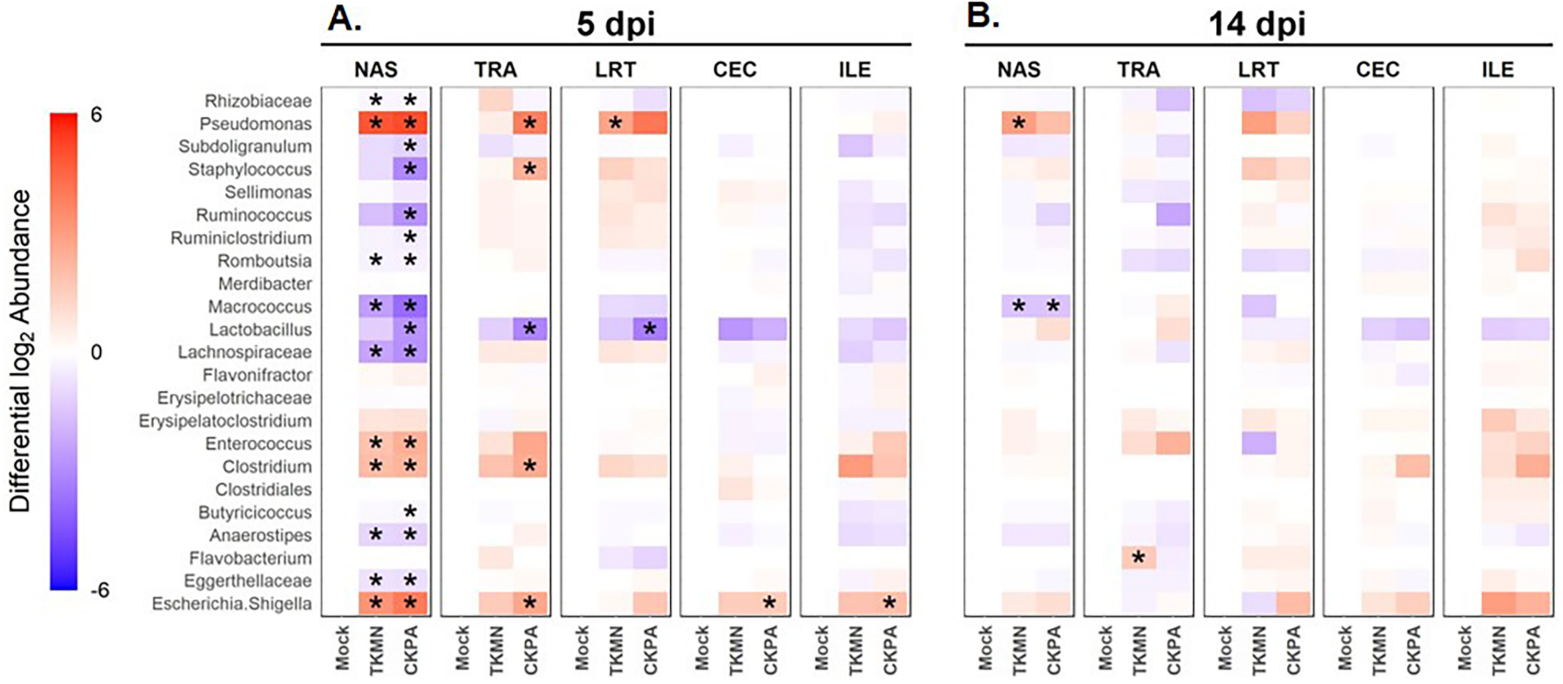
The abundance of predominant taxa of each body site increases or decreases in response to viral infection. Change in abundance is given by the ratio of mean relative abundance of a taxon for each virus-treated group to the mean relative abundance of the Mock group. The second log of this value gives the positive (red) or negative (blue) fold change of mean abundance in the AIV-treated groups from Mock. The intensity of the color indicates the magnitude of fold change in abundance of infected groups from the Mock. Stars indicate where abundance of predominant genera in AIV-treated groups was significantly different (*p* < 0.05) from the Mock. The Wilcoxon rank-sum test was used to determine significant differences in relative abundance. *P*-values were adjusted for multiple comparisons using the Holm method. See Table 2 for sample numbers/group. NAS = nasal cavity, TRA = trachea, LRT = lower respiratory tract, CEC = cecum, ILE = ileum.

Log_2_-transformed values of relative abundances were used to calculate differential abundance of the predominant bacteria between the Mock group and each of the virus-infected groups. As revealed by the heatmap in Fig. 4A, the abundances of several taxa were disrupted (increased or decreased) during active virus replication (5 dpi). For the CKPA group, 16 taxa were significantly disrupted in the nasal cavity, five in trachea, 1 in lower respiratory tract, one in ileum, and one in cecum. For the TKMN group, 10 taxa were significantly disrupted in the nasal cavity. Remarkably, the relative abundances of the 23 taxa were generally not significantly different between the infection groups (Fig. S2). Interestingly, bacteria from class Gammaproteobacteria were significantly disrupted across gut and respiratory sites; they tended to increase during active virus infection at 5 dpi (Fig. 4 and Fig. S3). Overall, the relative abundances of predominant genera and sample compositional variance explained by site were more similar between the three respiratory sites in CKPA group (more severe) compared to TKMN (less severe) and Mock (uninfected) groups (Fig. S4).

### Disruption of microbiota persists after virus clearance

**Figure 5:**
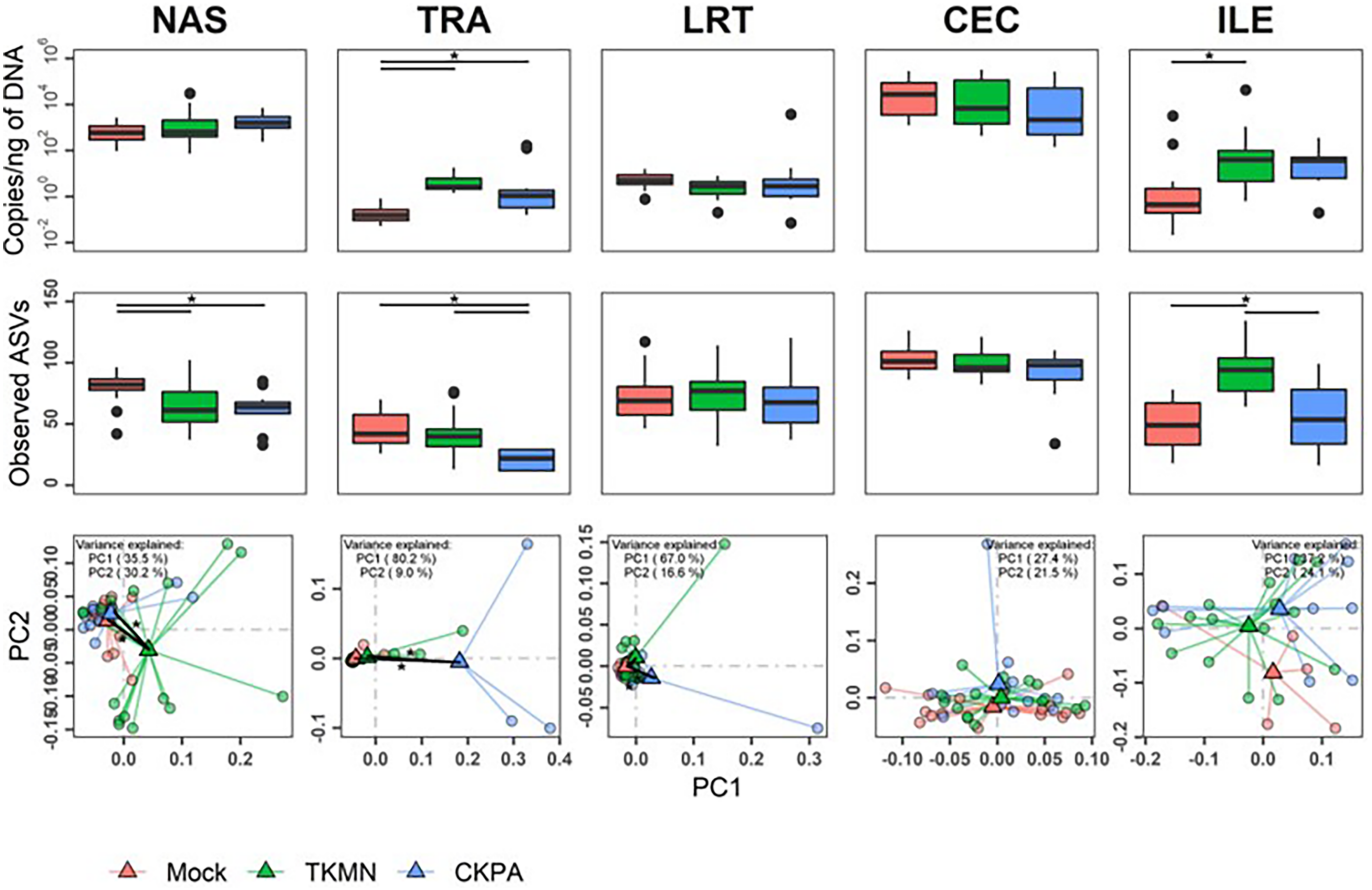
The density, diversity, and composition of the bacterial microbiome in the gut and respiratory tract at 14 days-post-infection. (A) Density is measured as the number of 16S rRNA gene copies present per nanogram (ng) of DNA used for sequencing. (B) Alpha diversity is measured as the number of distinct ASVs observed per sample after dataset rarefaction to 5,000 reads/sample. (C) Bacterial composition per sample is measured as the total abundance-weighted phylogenetic (UniFrac) distance between the bacteria of each sample. Black bars with stars indicate significant differences (*p* < 0.1) between groups. Significance in (A) and (B) observed with pairwise Wilcoxon rank sum test with Holm correction for multiple comparisons. Significance in (C) observed with pairwise PERMANOVA with Holm correction for multiple comparisons. See Table 2 for sample numbers/group. NAS = nasal cavity, TRA = trachea, LRT = lower respiratory tract, CEC = cecum, ILE = ileum.

After virus clearance at 14 dpi (Fig. 1A), the nasal bacterial content was restored to similar levels as the Mock group, but the ileal bacterial contents of the TKMN virus-infected birds remained significantly higher than the Mock group (Fig. 5A). Notably, in contrast to the earlier observation at 5 dpi (Fig. 3A), the virus infected groups showed a significant increase of bacterial content in the trachea (Fig. 5A).

Virus-induced species richness depletion was still evident in nasal cavities at 14 dpi. However, there was a dramatic increase in the number of observed ASVs in ilea of TKMN virus-infected birds (Fig. 5B), despite diminishment of virus-induced disruption of bacterial beta diversity to non-significant levels in the guts of both infected groups. Bacterial community compositions of TKMN virus-infected birds remained disrupted in nasal cavities and new disruptions were observed in lower respiratory tracts (Fig. 5C). Even though the relative abundances of virtually all predominant genera in virus-infected birds remained disrupted at 14 dpi, only 3 taxa were significantly disrupted by the TKMN virus (Fig. 4B).

### Microbiota disruption mainly correlates with local virus replication but not body weight

Taken together, the data presented above clearly show that influenza virus is responsible for the observed dysbiosis in all five body sites (Figs. 3, 4, 5, and Figs. S1–S3). Therefore, we performed a series of Spearman’s correlation tests to quantify the relationships between the abundance of site-associated predominant taxa and local viral titers (at 5 dpi) for the sites for which viral titers were measurable (excluding the lower respiratory tract; see Materials & Methods). Each infection group was compared with the Mock. The relative abundances of several taxa significantly correlated with viral titers depending on the body site.

**Figure 6:**
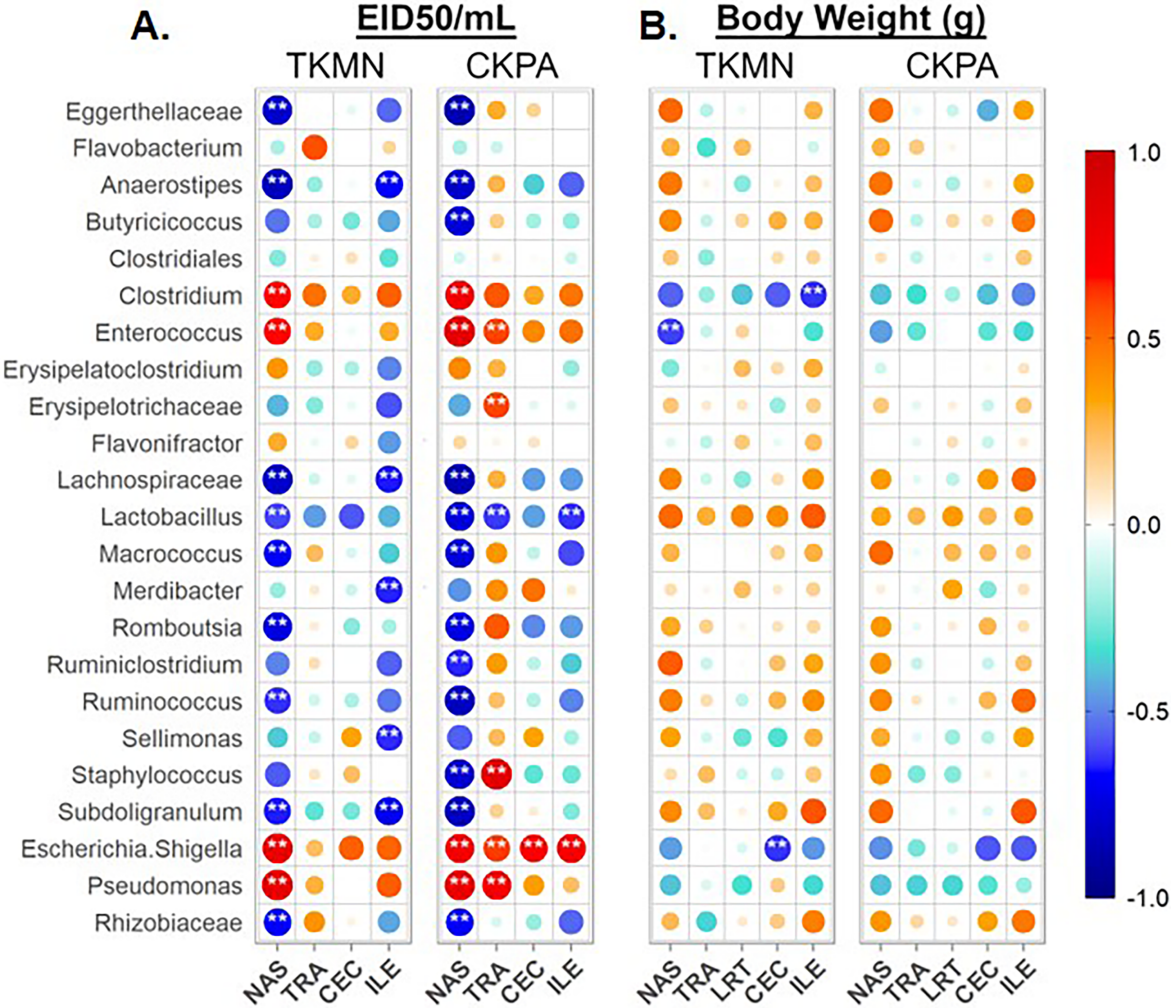
Spearman correlation between relative abundances of predominant taxa and virus titers (EID50/mL) and body weights (g) at 5 days-post-infection. (A) Correlation with virus titers. (B) Correlation with body weights. Spearman correlation strength is illustrated with both color and dot size, where larger, darker circles are stronger (—r— ≥ 0.5) and smaller, paler circles are weaker (—r— ≤ 0.5). Warm colors indicate positive correlation, cool colors indicate negative correlation, and white indicates no correlation. Stars indicate significance from *t*-test comparison, built into the ‘psych::corr.test’ function. One asterisk represents *p* < 0.1, two asterisks represent *p* < 0.05. *P*-values were adjusted for multiple comparisons using the Bonferroni method. See Table 2 for sample numbers/group. NAS = nasal cavity, TRA = trachea, LRT = lower respiratory tract, CEC = cecum, ILE = ileum.

In the nasal cavities, the abundances of unclassified Eggerthellaceae, *Flavobacterium*, unclassified Lachnospiraceae, *Lactobacillus*, *Macrococcus*, *Romboutsia*, *Ruminococcus*, *Subdoligranulum*, and unclassified Rhizobiaceae were negatively correlated with the titers of both TKMN and CKPA viruses, while those of *Clostridium*, *Enterococcus*, *Escherichia-Shigella*, and *Pseudomonas* were positively correlated (Fig. 6A). Further, *Butyricicoccus* and *Staphylococcus* abundances were negatively correlated with CKPA viral titers in the nasal cavities. Correlations observed in tracheas and ceca of TKMN virus-infected birds were generally weak and non-significant. However, in tracheas of CKPA infected birds, the abundances of *Enterococcus*, unclassified Erysipelotrichaceae, *Escherichia-Shigella*, *Staphylococcus*, and *Pseudomonas* were positively correlated with viral titers while *Lactobacillus* was negatively correlated. The abundances of *Anaerostipes*, unclassified Lachnospiraceae, *Merdibacter*, *Sellimonas*, and *Subdoligranulum* were negatively correlated with TKMN virus titers in the ileum. However, only *Escherichia-Shigella* (positive) and *Lactobacillus* (negative) were significantly correlated with ileal CKPA virus titers (Fig. 6A). Interestingly, despite the significant suppression of body weights in virus infected birds at 5 dpi (Fig. 2C), correlations between body weight and viral titers were generally weak and not statistically significant except for three correlations in nasal cavities, ceca, and ilea of TKMN virus-infected birds (Fig. 6B).

### Tracheal microbiota disruption correlates with innate immune gene expression

**Figure 7:**
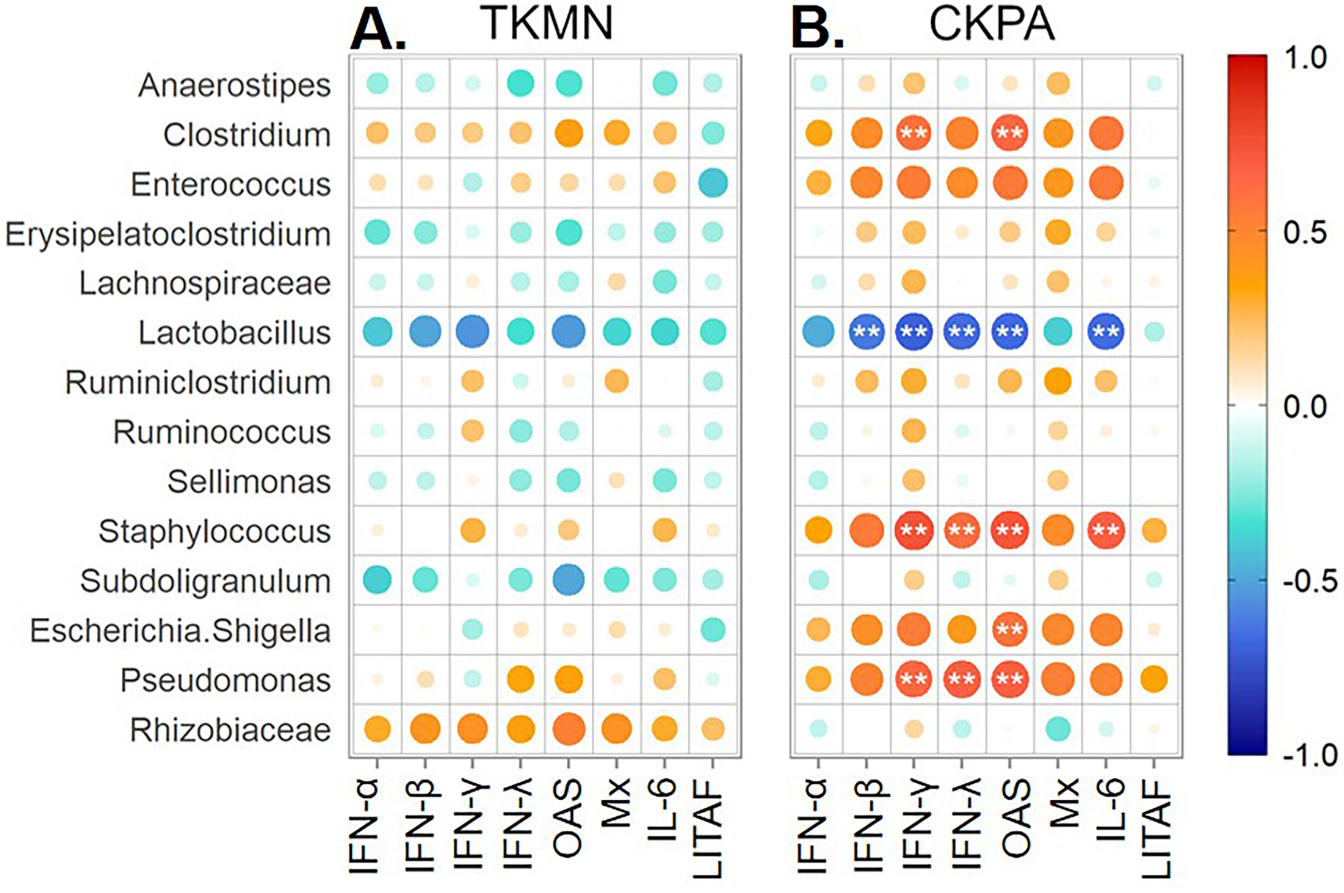
Spearman correlation between fold change in innate immune gene expression and relative abundances of predominant taxa in trachea at 5 days-post-infection. Each infected group was compared with the Mock. (A) TKMN group vs Mock. (B) CKPA group vs Mock. Spearman correlation strength is illustrated with both color and dot size, where larger, darker circles are stronger (—r— ≥ 0.5) and smaller, paler circles are weaker (—r— ≤ 0.5). Warm colors indicate positive correlation, cool colors indicate negative correlation, and white indicates no correlation. Stars indicate significance from t-test comparison, built into the ‘psych::corr.test’ function. One asterisk represents *p* < 0.1, two asterisks represent *p* < 0.05. *P*-values were adjusted for multiple comparisons using the Bonferroni method. See Table 2 for sample numbers/group. IFN = interferon. OAS = 2′–5′-oligoadenylate synthase, Mx = myxovirus (influenza virus) resistance 1, IL-6 = interleukin-6, LITAF = lipopolysaccharide-induced tumor necrosis factor-α factor.

Virus replication in trachea resulted in significant upregulation of IFN (α, β, γ, λ), IFN-stimulated (OAS and Mx), and IL-6 (proinflammatory) genes relative to mock-infected birds (Fig. 1B). Therefore, we paired the tracheal gene expression values (Log_2_ fold-change) with relative abundance values for predorminant bacterial taxa in the same birds euthanized at 5 dpi and performed Spearman’s correlation tests as described above. None of correlations in the TKMN group was statistically significant (Fig. 7A). However, the abundances of 5 taxa in the CKPA group were statistically correlated with the expression levels of one or more immune-related genes (Fig. 7B). As expected, because the LITAF gene expression is not stimulated by influenza virus infection (Fig. 1B and Fig. S1), its correlations with microbiota abundances were very weak and not significant (Fig. 7B). Of the IFN-related genes examined in the CKPA group, IFN-α and Mx were poorly correlated with dysbiosis, again reflecting the low levels detected at 5 dpi. Upregulation of IFN-γ, IFN-λ and OAS genes was correlated with significant increase in the abundance of *Clostridium*, *Pseudomonas*, and *Staphylococcus*, and a significant decrease in *Lactobacillus*. Additionally, OAS gene upregulation was correlated with significant increase in the abundance of *Escherichia-Shigella*, while the expression of IL-6 proinflammatory gene correlated with significant increase of *Staphylococcus* and depletion of *Lactobacillus* (Fig. 7B).

## Discussion

We have confirmed previous phenotypic observations on differential replication and pathogenicity between the two H5N2 viruses (Pillai et al., 2010a), herein denoted CKPA and TKMN. Further, we newly identified additional distinguishable phenotypes regarding bursal atrophy relative to uninfected birds, expression of innate immune genes, and biotic disruption of commensal gut and respiratory microbiota.

As observed before (Pillai et al., 2010a), the CKPA virus replicated to higher titers in the URT and induced more severe clinical signs in poults. However, viral titers in the gut (ileum and cecum) were statistically indistinguishable between the viruses in this study, which contrasts the higher cloacal virus shedding observed with CKPA virus in our earlier report (Pillai et al., 2010a). This discrepancy may be reflective of the larger sample size used in this study (*n* = 16) relative to the previous study (*n* = 9 or 6). Nevertheless, the TKMN virus induced bursal atrophy at 5 dpi as observed with other LPAI viruses in chickens without clinical signs (Jackwood et al., 2010) or those displaying signs such as depression, puffing, edema of face and head, conjunctivitis, ruffled feathers, tracheal rales, and diarrhea (Abdel Hamid et al., 2016; Hadipour et al., 2011). Yet the bursa body weight ratios of a few individuals in the CKPA group were significantly below the average ratio for the group, suggesting that bursal atrophy induction in turkeys is independent of clinical signs. Nevertheless, the more severe induction of bursal atrophy by the TKMN virus corresponded with reduction of HI antibodies, suggesting that, like the infectious bursal disease virus (Withers, Young & Davison, 2005), LPAI viruses may suppress systemic antibody responses in turkeys by destroying bursal follicles resulting in B-cells depletion. Since LPAI viruses isolated from turkeys are associated with severe clinical symptoms (Jimenez-Bluhm et al., 2019; Kleven, Nelson & Deshmukh, 1970; Reid et al., 2016; Zanella, Dall’Ara & Martino, 2001), the differential induction of clinical signs and pathological lesions between CKPA and TKMN viruses in this study is unlikely due to differences in adaptation in turkeys. This needs to be investigated further.

Differential replication rates between the two viruses resulted in corresponding levels of LPAI virus-induced innate immune gene expression in the tracheas of young turkeys. While this might be the first account of innate immune gene expression in response to LPAI virus infection in turkeys, other turkey studies have shown cytokine and chemokine gene upregulation following pathogen exposures. In ceca of turkeys infected with the protozoa *Eimeria adenoeides*, Gadde et al. (2011) observed increases in the expression of CXCLi2 chemokine gene at 4 and 7 dpi, IFN-γ gene at 4 dpi, and IL1 β and IL13 genes at 7 dpi. Another protozoa study (Powell et al., 2009) showed that while chickens were able to mount an effective cecal innate response (with increased IL1-β, CXCLi2 and IL-6 gene expression) within 1-6 dpi and prevent *Histomonas meleagridis* from migrating to the liver, turkeys were not. Moreover, *in ovo* infection with avian metapneumovirus subtype C resulted in significant increase in IFN-γ and IL-10 gene expression in URT at 4 dpi (day of hatch) and 9 dpi (5 days post-hatch) (Cha et al., 2011). We speculate that a similar upregulation of IFNs and proinflammatory cytokines may be induced in the nasal cavities, ilea, and ceca of LPAI virus-infected turkeys in correlation with the level of virus replication.

Another consequence of virus infection is modulation of commensal microbiota locally at the site of virus replication or systemically (Borges et al., 2018; Deriu et al., 2016; Ganz et al., 2017; Hird et al., 2018; Li et al., 2018; Wen et al., 2018; Yitbarek et al., 2018c; Zhao et al., 2018). Here, we observed a loss of species diversity due to the overgrowth of certain local taxa during infection with LPAI virus, exemplified in some sites by a negative relationship between bacterial content and species richness. An increase in the absolute numbers of certain bacterial species may lead to decreases in relative abundances but not the absolute numbers of other bacterial species. We will clarify this in the future through qPCR using primers targeting specific bacterial families.

There has been a knowledge gap in the interactions between influenza virus and respiratory microbiota in avian species, which our study has attempted to fill. We found the strongest microbiota disruption in the nasal cavity followed by trachea and lower respiratory tract, respectively. The level of respiratory microbiota disruption (enrichment or depletion) was strain specific, with CKPA virus causing significant disruptions mostly in the nasal cavity, followed by the trachea and lower respiratory tract, while the TKMN virus was disruptive only in the nasal cavity. These differential changes in bacterial abundance may arise from the effects of specific cytokines induced by the virus as discussed further below. Nevertheless, bacterial compositions of the three respiratory sites were distinct from one another in healthy birds, but virus-induced dysbiosis resulted in greater compositional similarity between sites. The increase in site connectivity between healthy and diseased states has been proposed in humans (Dickson et al., 2015; Proctor & Relman, 2017) but has not been observed in avian species. Nevertheless, even during acute upper respiratory infection, the nasal cavity microbiota remains distinct from that of lower respiratory sites. This has also been observed with human lung models, in which lung microbiota are more similar to oral cavity or tracheal microbiota than nasal cavity microbiota under both healthy and diseased conditions (Bassis et al., 2015; Boutin et al., 2015; Charlson et al., 2011; Segal et al., 2013).

While there were subtle differences in enrichment/depletion of bacterial genera between the respiratory and digestive systems following virus infection, at class level, Gammaproteobacteria (such as *Escherichia-Shigella*) tended to be enriched, especially in CKPA virus-infected birds. Overgrowth of bacteria in phylum Proteobacteria was previously observed in gut microbiota of chickens infected with influenza virus via the oral-nasal route (Li et al., 2018; Yitbarek et al., 2018c). It is currently unknown if Gammaproteobacteria are enriched in turkeys infected by pathogens other than LPAI virus.

Interestingly, subtle signals of dysbiosis persisted in both the gut and respiratory tract after virus clearance. However, the magnitudes of diversity and relative abundance disruptions were greatly reduced in turkeys by 14 dpi. Post-viral-shedding persistence of dysbiosis in other avian species is yet to be documented, although time-course experiments with chickens infected with LPAI H9N2 viruses have shown continuous decline in the level of gut microbiota disruption with diminishment of cloacal virus shedding (Li et al., 2018; Yitbarek et al., 2018c). Moreover, mice infected with an LPAI H5N1 virus also experienced transient dysbiosis of gut and respiratory microbiota, which peaked during active virus replication at 7 dpi and normalized after virus clearance at 14 dpi (Yildiz et al., 2018). Regardless of how long it persists, virus-induced dysbiosis can trigger full blown infection with potential pathogens that may be lurking in poultry flocks at subclinical levels (Ngunjiri et al., 2019b; Taylor et al., 2020). This is in line with co-infection studies in turkeys and chickens showing that influenza virus and bacterial pathogens (e.g., Proteobacteria like *E. coli* and *Salmonella*) can not only enhance the replication of each other but also exacerbate the clinical outcomes and mortality rates (Barbour et al., 2009; Deriu et al., 2016; Mosleh et al., 2017; Taha et al., 2019; Umar et al., 2018), and potentially lead to higher economic losses.

Different mechanisms of virus-induced dysbiosis may operate at different body sites, with one such mechanism being cytokine production. In mice co-infected with a human H1N1 influenza virus and *Streptococcus pneumoniae*, Duvigneau and colleagues (Duvigneau et al., 2016) showed that the outgrowth of *S. pneumoniae* was promoted by IFN-γ and antagonized by IL-6. Further, **in vivo** neutralization of influenza virus-induced IFN-γ with antibodies resulted in enhancement of local bacterial clearance in the lungs, validating the role for this cytokine in promoting *S. pneumoniae* (Sharma-Chawla et al., 2019). Another study showed that *S. pneumoniae* outgrowth in mice is also induced by IL-20 and blocked by treatment with an antibody against IL-20 receptor subunit beta (Madouri et al., 2018). The pro-inflammatory cytokine-mediated mechanism of bacterial growth augmentation involves a direct interaction between cytokines and bacterial cells. For example, addition of TNF-α, IL-1 β, IL-6, IL-8 and IFN-γ cytokines (from commercial sources) in bacterial growth medium triggered a rapid growth of uropathogenic *E. coli* strain CFT073, possibly by increasing iron acquisition by the bacterium (Engelsoy, Rangel & Demirel, 2019). Type I IFNs are also directly involved in dysbiosis induction. In a co-infection study using IFN α/β receptor knockout mice, Lee and co-workers (Lee et al., 2015) found influenza virus-induced type I IFN to enhance susceptibility to *E. coli* and *Staphylococcus aureus*. Therefore, cytokines are key mediators of dysbiosis including that induced by influenza virus.

In non-infected maturing chicken broilers, Oakley and Kogut (Oakley & Kogut, 2016) reported that the expression of pro-inflammatory cytokine genes (IL-18, IL-1β, and IL-6) was generally correlated with decreased Firmicutes abundance and increased Proteobacteria abundance (Oakley & Kogut, 2016). In tracheas of CKPA virus-infected turkeys, we found that expression of type III IFN (IFN-λ) gene mirrors that of types I and II IFNs (IFN-α/β and IFN-γ, respectively) genes regarding the association with tracheal microbiota disruption: outgrowth of Firmicutes (*Staphylococcus*, *Clostridium*, and *Enterococcus*) and Proteobacteria (*Pseudomonas* and *Escherichia-Shigella*), and inhibition of Firmicutes (*Lactobacillus*). Likewise, expression of IL-6 was associated with significant *Staphylococcus* outgrowth and *Lactobacillus* depletion. Further studies in knockout avian models of innate immune genes are needed to determine the causal relationships between cytokine gene expression and commensal bacteria compositions in different body sites, to facilitate the development of intervention strategies such as probiotics-based microbiota modulation.

Another plausible cause of the observed dysbiosis is virus-induced changes in nutritional intake. While feed intake was not measured in this study, at 5 dpi, there was significant suppression of body weights in virus infected birds relative to the Mock group. However, correlations between body weight and viral titers were generally weak and not statistically significant, suggesting that reduced feed intake and, therefore, body weight suppression may not explain gut microbiota disruption in infected birds. Furthermore, it will be interesting to link changes in nutrition intake to respiratory tract dysbiosis in LPAI virus-infected birds.

Despite the interesting findings of this study, we note the limitation of our experimental design. For examples, we did not collect data to assess how the extent of mucosal barrier injury (Li et al., 2018) in gut and respiratory sites affected the observed associations among microbiota, influenza virus titers, and innate gene expression profiles. Furthermore, the expression of IFN-α/β/λ and IL-6 genes at high levels may not indicate the production of high amounts of functional proteins, as demonstrated in our recent work where a subpopulation of avian influenza virus blocked the synthesis of IFN-β protein without inhibiting the transcription of IFN-β mRNA (Ghorbani et al., 2020). Moreover, without bacterial metabolite or transcriptome data, it remains unknown if the alteration of bacterial diversity, and abundance and following virus infection in this study indicated functional changes of microbiota. This study also did not answer question whether the level of LPAI virus-induced disease can be predicted by a priori microbiota composition or be exacerbated by outgrowth of secondary pathogens.

## Conclusions

The data presented in this study have uncovered the effects of LPAI H5N2 virus infection and dysbiosis in the digestive and respiratory tracts of turkeys. LPAI virus-induced dysbiosis in turkeys is transient but it persists for some time after viral clearance. The most dramatic impacts on microbiota during active virus replication were observed in the nasal cavity and ileum, where β-diversity was disturbed, and species diversity decreased as the number of bacteria (bacterial content) increased. While the study design did not allow determination of causal effects between cytokine gene expression and microbiota disruption, mediator cytokines are likely involved in the biotic disruption mechanism in LPAI virus-infected turkeys. Other studies have reported disruption of gut bacteria diversity (change in dissimilarity and species extinction) in several avian species infected with LPAI viruses, but LPAI virus-induced effects on avian respiratory microbiota have yet to be examined for species other than turkeys.

## Supplemental Information

10.7717/peerj.11806/supp-1 Supplemental Information 1 Correlation between viral titers and fold-change in innate immune gene expression at 5 days post-infection See Table 2 for sample numbers/group. IFN = interferon. OAS = 2′–5′-oligoadenylate synthase, Mx = myxovirus (influenza virus) resistance 1, IL-6 = interleukin-6, LITAF = lipopolysaccharide-induced tumor necrosis factor-α factor.

10.7717/peerj.11806/supp-2 Supplemental Information 2 Comparison of the abundance of predominant taxa of each body site between the infected groups (A) 5 days-post-infection. (B) 14 days-post-infection. Change in abundance is given by the ratio of mean relative abundance of a taxon for TKMN group to the mean relative abundance of the CKPA group. The Log2 of this value gives the positive (red) or negative (blue) fold change of mean abundance in the TKMN group from CKPA group. The intensity of the color indicates the magnitude of fold change in abundance of infected groups from the Mock. Stars indicate where abundance of predominant genera in one infected group was significantly different (*p* < 0.05) from the other group. The Wilcoxon rank-sum test was used to determine significant differences in relative abundance. *P*-values were adjusted for multiple comparisons using the Holm method. See Table 2 for sample numbers/group. NAS = nasal cavity, TRA = trachea, LRT = lower respiratory tract, CEC = cecum, ILE = ileum.

10.7717/peerj.11806/supp-3 Supplemental Information 3 The total relative abundance of class Gammaproteobacteria for each sample collected at 5 and 14 days post infection The black crossbar indicates the mean relative abundance for the group. Abundance differences between groups were tested for significance (*p* < 0.05) using a pairwise Wilcoxon rank sum test and Holm correction for multiple comparisons. Significant differences are indicated by starred bars at the top of each panel. See Table 2 for sample numbers/group. NAS = nasal cavity, TRA = trachea, LRT = lower respiratory tract, CEC = cecum, ILE = ileum. dpi = days post-infection.

10.7717/peerj.11806/supp-4 Supplemental Information 4 Comparison of predominant bacterial profiles in the respiratory tract according to infection status at 5 days post infection (A) Bar chart profiles show total relative abundance of predominant genera in each site according to treatment group. Stacked bars are colored by genus and indicate consensus values of relative abundance for a genus from all samples of a site (*x*-axis), the total relative abundance of which is 1. (B) Variance explained (R2) by grouping samples by site in pairwise comparison with PERMANOVA (‘pairwiseAdonis::pairwise.adonis’) with Holm correction for multiple comparisons. Sample distances were calculated using the Bray-Curtis distance algorithm with a sample-by-genus matrix of relative abundances. Cells shaded red indicate more sample compositional variance explained by site, and therefore greater compositional difference between sites. Numbers in bold indicate significant differences (*p* < 0.05) according to pairwise PERMANOVA. See Table 2 for sample numbers/group. NAS = nasal cavity, TRA = trachea, LRT = lower respiratory tract, CEC = cecum, ILE = ileum.

10.7717/peerj.11806/supp-5 Supplemental Information 5 Location, identity, and number of predominant taxa

10.7717/peerj.11806/supp-6 Supplemental Information 6 PERMANOVA results for principal coordinates

10.7717/peerj.11806/supp-7 Supplemental Information 7 Raw qRT-PCR data

10.7717/peerj.11806/supp-8 Supplemental Information 8 Arrive guidelines

## Additional Information and Declarations

### Data Availability

The following information was supplied regarding data availability: Raw data files and metadata are available at NCBI BioProject database PRJNA644054. Raw data files are also available at the Sequence Read Archive (SRA): SAMN15446589–SAMN15447100. Pipe scripts for QIIME2 sequence processing and generation of the ASV table and R scripts for analysis are publicly available at GitHub: https://github.com/kjmtaylor22/cwl-cats.
