# Supplementary figures and images for: Influenza A virus infection in turkeys induces respiratory and enteric bacterial dysbiosis correlating with cytokine gene expression
Source: PeerJ. 2021 Jul 22;9:e11806. doi: 10.7717/peerj.11806 (PMC8310620; doi:10.7717/peerj.11806)

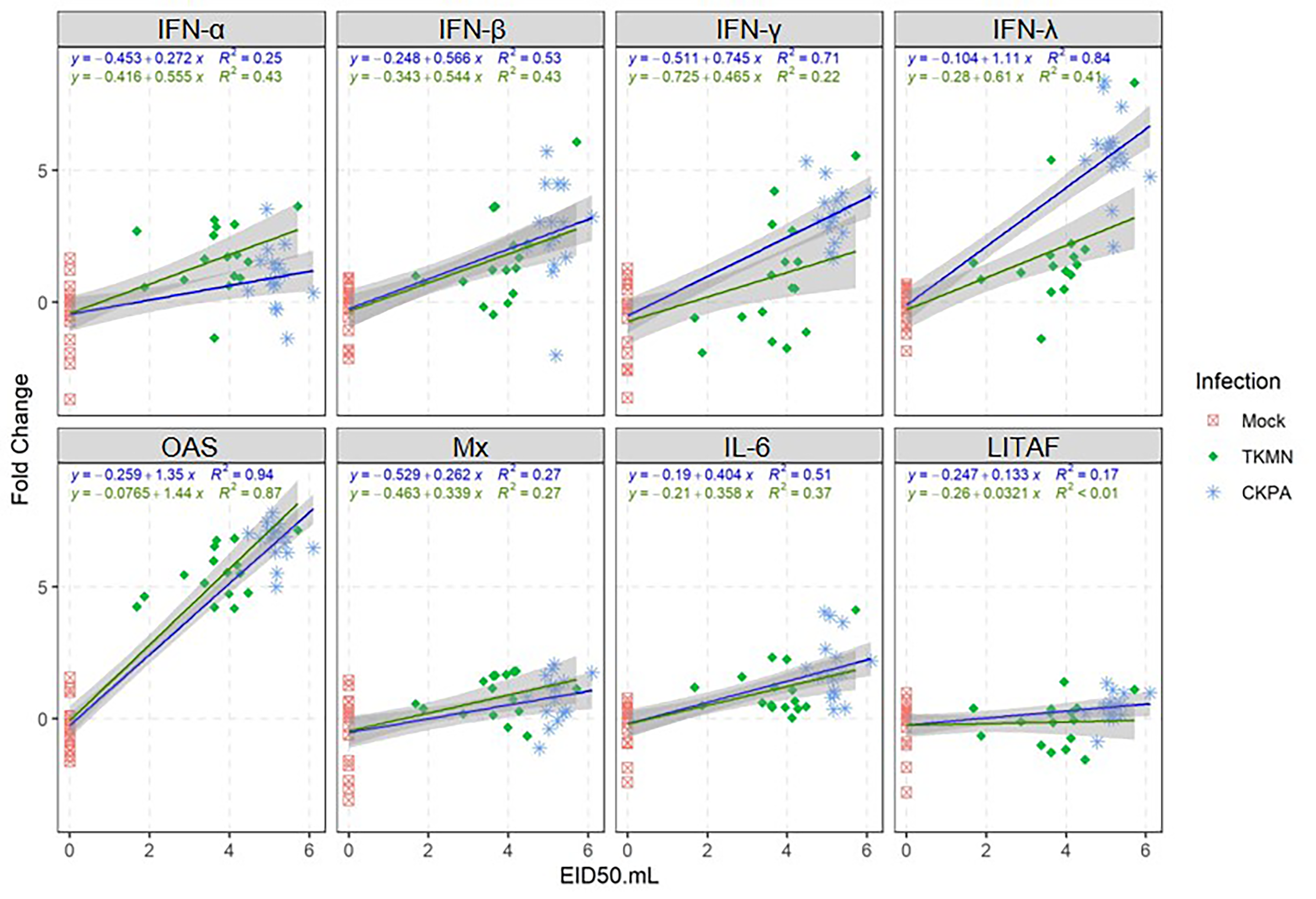

Supplement: Supplemental Information 1 — See Table 2 for sample numbers/group. IFN = interferon. OAS = 2′–5′-oligoadenylate synthase, Mx = myxovirus (influenza virus) resistance 1, IL-6 = interleukin-6, LITAF = lipopolysaccharide-induced tumor necrosis factor-α factor. [file peerj-09-11806-s001.png]

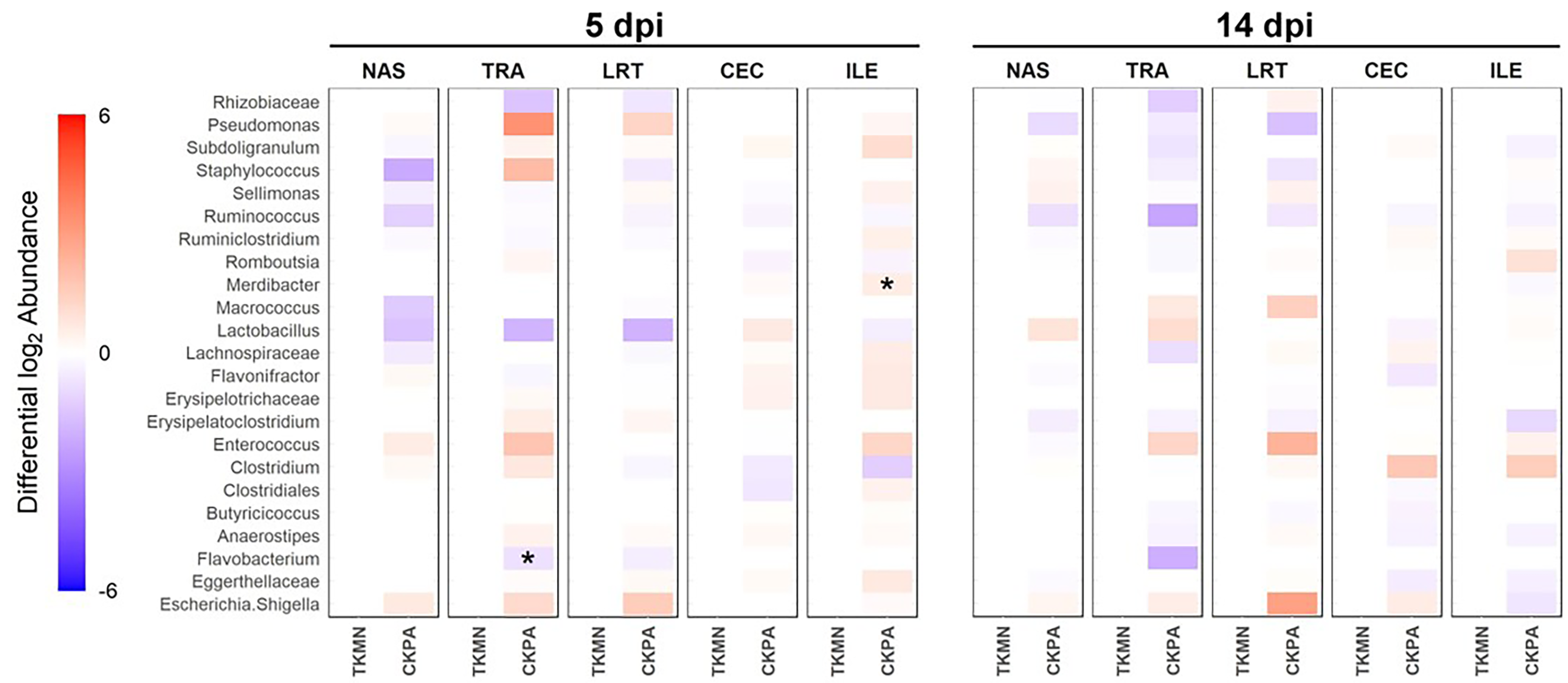

Supplement: Supplemental Information 2 — (A) 5 days-post-infection. (B) 14 days-post-infection. Change in abundance is given by the ratio of mean relative abundance of a taxon for TKMN group to the mean relative abundance of the CKPA group. The Log2 of this value gives the positive (red) or negative (blue) fold change of mean abundance in the TKMN group from CKPA group. The intensity of the color indicates the magnitude of fold change in abundance of infected groups from the Mock. Stars indicate where abundance of predominant genera in one infected group was significantly different (p < 0.05) from the other group. The Wilcoxon rank-sum test was used to determine significant differences in relative abundance. P-values were adjusted for multiple comparisons using the Holm method. See Table 2 for sample numbers/group. NAS = nasal cavity, TRA = trachea, LRT = lower respiratory tract, CEC = cecum, ILE = ileum. [file peerj-09-11806-s002.png]

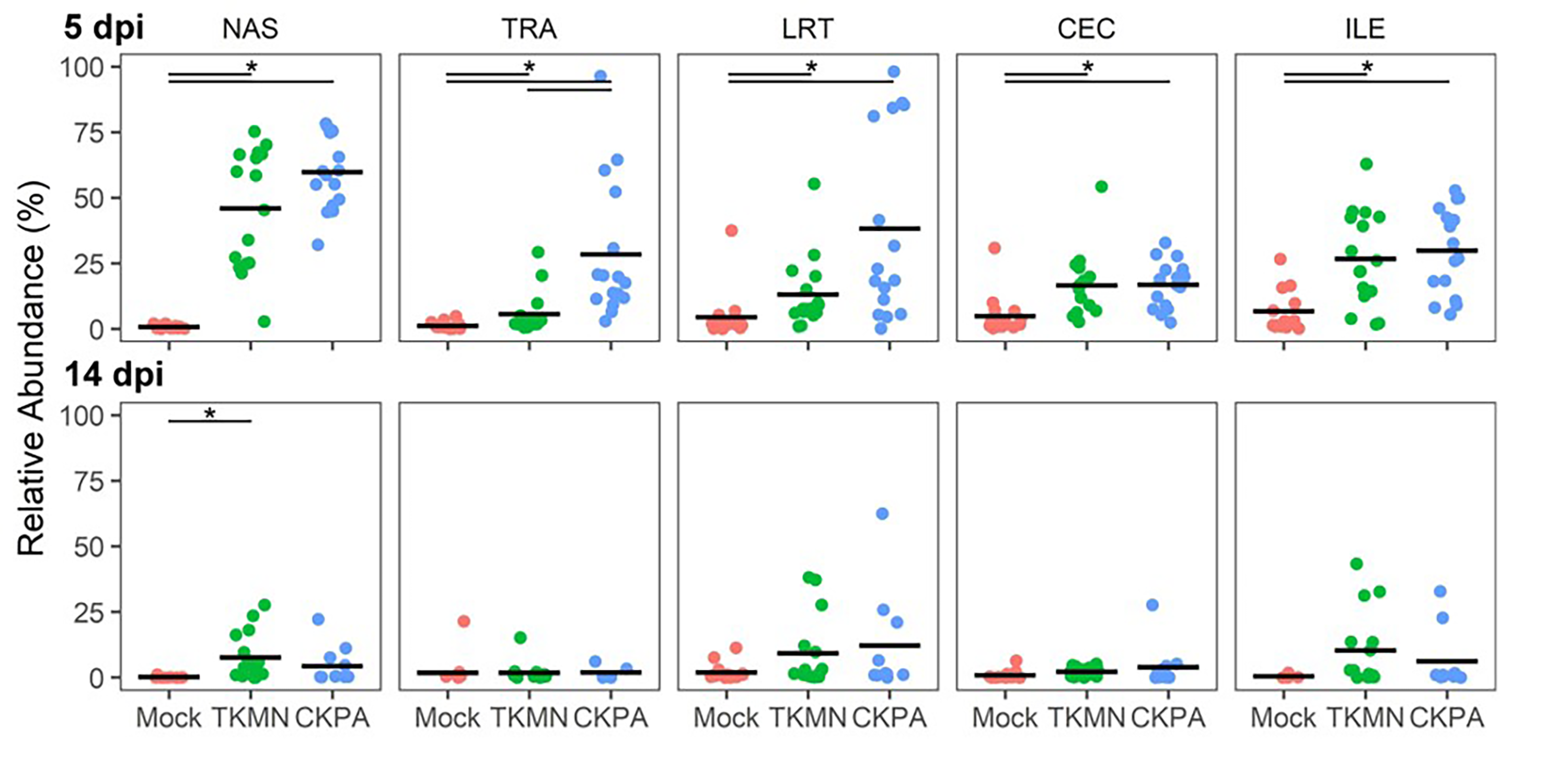

Supplement: Supplemental Information 3 — The black crossbar indicates the mean relative abundance for the group. Abundance differences between groups were tested for significance (p < 0.05) using a pairwise Wilcoxon rank sum test and Holm correction for multiple comparisons. Significant differences are indicated by starred bars at the top of each panel. See Table 2 for sample numbers/group. NAS = nasal cavity, TRA = trachea, LRT = lower respiratory tract, CEC = cecum, ILE = ileum. dpi = days post-infection. [file peerj-09-11806-s003.png]

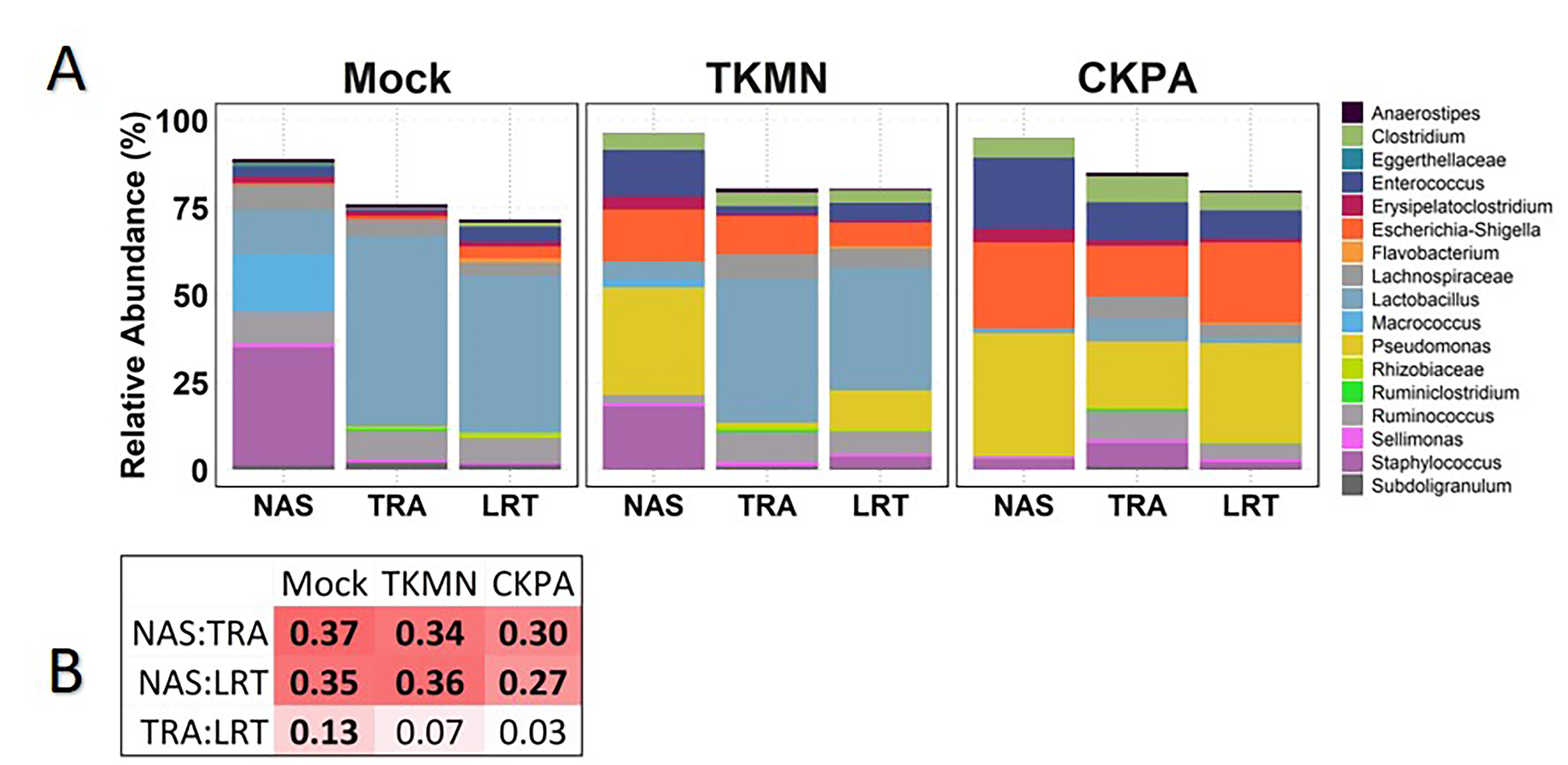

Supplement: Supplemental Information 4 — (A) Bar chart profiles show total relative abundance of predominant genera in each site according to treatment group. Stacked bars are colored by genus and indicate consensus values of relative abundance for a genus from all samples of a site (x-axis), the total relative abundance of which is 1. (B) Variance explained (R2) by grouping samples by site in pairwise comparison with PERMANOVA (‘pairwiseAdonis::pairwise.adonis’) with Holm correction for multiple comparisons. Sample distances were calculated using the Bray-Curtis distance algorithm with a sample-by-genus matrix of relative abundances. Cells shaded red indicate more sample compositional variance explained by site, and therefore greater compositional difference between sites. Numbers in bold indicate significant differences (p < 0.05) according to pairwise PERMANOVA. See Table 2 for sample numbers/group. NAS = nasal cavity, TRA = trachea, LRT = lower respiratory tract, CEC = cecum, ILE = ileum. [file peerj-09-11806-s004.png]
