# Supplementary material for: Influenza A virus infection in turkeys induces respiratory and enteric bacterial dysbiosis correlating with cytokine gene expression
Source: PeerJ. 2021 Jul 22;9:e11806. doi: 10.7717/peerj.11806 (PMC8310620; doi:10.7717/peerj.11806)
Supplement: Supplemental Information 5 [file peerj-09-11806-s005.docx]

| Table S1. Location, identity, and number of predominant taxa | | | | | | | | | | | | | | | | | |
| --- | --- | --- | --- | --- | --- | --- | --- | --- | --- | --- | --- | --- | --- | --- | --- | --- | --- |
| Lowest taxonomic classification | Taxon name | # ASVs | Nasal | | | Trachea | | | LRT | | | Cecum | | | Ileum | | |
|  |  |  | Mock | TKMN | CKPA | Mock | TKMN | CKPA | Mock | TKMN | CKPA | Mock | TKMN | CKPA | Mock | TKMN | CKPA |
| Genus | Anaerostipes | 3 | × |  |  |  |  |  |  |  |  | × | × | × | × |  |  |
|  | Butyricicoccus | 6 |  |  |  |  |  |  |  |  |  | × | × | × |  |  |  |
|  | Clostridium | 38 |  |  | × |  |  |  |  | × | × |  |  |  |  | × |  |
|  | Enterococcus | 5 |  | × | × |  |  |  |  | × | × |  |  |  |  |  |  |
|  | Erysipelatoclostridium | 11 | × | × |  | × |  |  |  |  |  | × | × | × | × |  | × |
|  | Escherichia-Shigella | 2 |  | × | × |  | × | × |  |  | × | × | × | × | × | × | × |
|  | Flavobacterium | 13 |  |  |  |  |  |  | × |  |  |  |  |  |  |  |  |
|  | Flavonifractor | 2 |  |  |  |  |  |  |  |  |  | × | × | × |  |  |  |
|  | Lactobacillus | 21 | × |  |  | × | × |  | × |  |  |  |  |  | × |  |  |
|  | Macrococcus | 1 | × | × |  |  |  |  |  |  |  |  |  |  |  |  |  |
|  | Merdibacter | 1 |  |  |  |  |  |  |  |  |  | × | × | × |  |  |  |
|  | Pseudomonas | 19 |  | × | × |  |  |  |  |  |  |  |  |  |  |  |  |
|  | Romboutsia | 10 |  |  |  |  |  |  |  |  |  |  |  |  | × |  |  |
|  | Ruminiclostridium | 14 |  |  |  |  |  |  |  |  |  | × | × | × |  |  |  |
|  | Ruminococcus | 23 | × |  |  |  | × |  |  |  |  | × | × | × | × |  |  |
|  | Sellimonas | 7 |  |  |  |  |  |  |  |  |  | × |  | × |  |  |  |
|  | Staphylococcus | 7 | × | × | × |  |  | × |  |  |  |  |  |  |  |  |  |
|  | Subdoligranulum | 4 | × |  |  | × |  |  |  |  |  | × |  |  | × |  | × |
| Family | Eggerthellaceae | 1 |  |  |  |  |  |  |  |  |  | × | × | × |  |  |  |
|  | Erysipelotrichaceae | 1 |  |  |  |  |  |  |  |  |  |  |  |  |  |  |  |
|  | Lachnospiraceae | 68 | × |  |  |  |  |  |  |  |  | × | × | × | × |  |  |
|  | Rhizobiaceae | 7 |  |  |  |  |  |  | × |  |  |  |  |  |  |  |  |
| Order | Clostridiales | 14 |  |  |  |  |  |  |  |  |  |  | × | × |  |  |  |
| Total: | 23 | 278 | 8/23 | 6/23 | 5/23 | 3/23 | 3/23 | 2/23 | 3/23 | 2/23 | 3/23 | 12/23 | 11/23 | 12/23 | 8/23 | 2/23 | 3/23 |
