# Supplementary material for: Influenza A virus infection in turkeys induces respiratory and enteric bacterial dysbiosis correlating with cytokine gene expression
Source: PeerJ. 2021 Jul 22;9:e11806. doi: 10.7717/peerj.11806 (PMC8310620; doi:10.7717/peerj.11806)
Supplement: Supplemental Information 6 [file peerj-09-11806-s006.docx]

| Supplementary Table 2: PERMANOVA results for principal coordinates | | | | | | | | | | |
| --- | --- | --- | --- | --- | --- | --- | --- | --- | --- | --- |
| DPI | Body Site | Pairs | | Df | SumsOfSqs | F.Model | R2 | p.value | p.adjusted | significance |
| 5 | NAS | Mock | CKPA | 1 | 1.563 | 246.769 | 0.892 | 0.001 | 0.003 | * |
|  |  | Mock | TKMN | 1 | 0.874 | 46.289 | 0.607 | 0.001 | 0.003 | * |
|  |  | CKPA | TKMN | 1 | 0.123 | 6.304 | 0.174 | 0.01 | 0.03 | * |
|  | TRA | Mock | CKPA | 1 | 0.044 | 0.872 | 0.028 | 0.341 | 1 |  |
|  |  | Mock | TKMN | 1 | 0.108 | 1.977 | 0.062 | 0.157 | 0.471 |  |
|  |  | CKPA | TKMN | 1 | 0.048 | 1.134 | 0.036 | 0.289 | 0.867 |  |
|  | LRT | Mock | CKPA | 1 | 0.031 | 0.582 | 0.019 | 0.501 | 1 |  |
|  |  | Mock | TKMN | 1 | 0.024 | 0.417 | 0.014 | 0.536 | 1 |  |
|  |  | CKPA | TKMN | 1 | 0.065 | 1.442 | 0.046 | 0.251 | 0.753 |  |
|  | CEC | Mock | CKPA | 1 | 0.107 | 9.376 | 0.238 | 0.001 | 0.003 | * |
|  |  | Mock | TKMN | 1 | 0.058 | 3.954 | 0.116 | 0.007 | 0.021 | * |
|  |  | CKPA | TKMN | 1 | 0.018 | 1.421 | 0.045 | 0.236 | 0.708 |  |
|  | ILE | Mock | CKPA | 1 | 0.171 | 10.236 | 0.275 | 0.001 | 0.003 | * |
|  |  | Mock | TKMN | 1 | 0.270 | 15.091 | 0.359 | 0.001 | 0.003 | * |
|  |  | CKPA | TKMN | 1 | 0.036 | 1.638 | 0.052 | 0.191 | 0.573 |  |
| 14 | NAS | Mock | CKPA | 1 | 0.029 | 1.740 | 0.068 | 0.167 | 0.501 |  |
|  |  | Mock | TKMN | 1 | 0.334 | 13.539 | 0.311 | 0.001 | 0.003 | * |
|  |  | CKPA | TKMN | 1 | 0.185 | 5.778 | 0.194 | 0.013 | 0.039 | * |
|  | TRA | Mock | CKPA | 1 | 0.433 | 9.811 | 0.366 | 0.019 | 0.057 |  |
|  |  | Mock | TKMN | 1 | 0.079 | 2.203 | 0.073 | 0.134 | 0.402 |  |
|  |  | CKPA | TKMN | 1 | 0.261 | 3.079 | 0.153 | 0.057 | 0.171 |  |
|  | LRT | Mock | CKPA | 1 | 0.116 | 4.345 | 0.159 | 0.001 | 0.003 | * |
|  |  | Mock | TKMN | 1 | 0.095 | 5.084 | 0.149 | 0.001 | 0.003 | * |
|  |  | CKPA | TKMN | 1 | 0.029 | 0.627 | 0.028 | 0.547 | 1 |  |
|  | CEC | Mock | CKPA | 1 | 0.011 | 0.619 | 0.025 | 0.671 | 1 |  |
|  |  | Mock | TKMN | 1 | 0.015 | 1.335 | 0.043 | 0.234 | 0.702 |  |
|  |  | CKPA | TKMN | 1 | 0.013 | 0.789 | 0.032 | 0.576 | 1 |  |
|  | ILE | Mock | CKPA | 1 | 0.022 | 1.028 | 0.079 | 0.328 | 0.984 |  |
|  |  | Mock | TKMN | 1 | 0.034 | 2.000 | 0.100 | 0.106 | 0.318 |  |
|  |  | CKPA | TKMN | 1 | 0.039 | 2.114 | 0.088 | 0.092 | 0.276 |  |
